# p53 inhibits CTR1-mediated cisplatin absorption by suppressing SP1 nuclear translocation in osteosarcoma

**DOI:** 10.3389/fonc.2022.1047194

**Published:** 2023-01-26

**Authors:** Lei Yong, Yan Shi, Hai-Long Wu, Qi-Yuan Dong, Jing Guo, Li-Sheng Hu, Wen-Hao Wang, Zhi-Ping Guan, Bin-Sheng Yu

**Affiliations:** ^1^ Shenzhen Key Laboratory of Spine Surgery, Department of Spine Surgery, Peking University Shenzhen Hospital, Shenzhen, China; ^2^ Shenzhen Engineering Laboratory of Orthopaedic Regenerative Technologies, National & Local Joint Engineering Research Center of Orthopaedic Biomaterials, Peking University Shenzhen Hospital, Shenzhen, China

**Keywords:** osteosarcoma, cisplatin, p53, SP1, CTR1

## Abstract

**Background:**

Osteosarcoma (OS) is a malignant bone tumor mainly affecting children and young adolescents. Cisplatin is a first-line chemotherapy drug for OS, however, drug resistance severely limits the survival of OS. Nevertheless, cellular factors in cisplatin resistance for OS remain obscure. In this study, the function and potential mechanism of p53 in cisplatin absorption were explored in OS cells.

**Methods:**

The CRISPR-Cas9 gene editing technology was performed to obtain p53 gene knock-out U2OS cells. The p53 over-expression 143B cell line was established by lentivirus-mediated virus infection. Moreover, the functions of p53 and CTR1 in cisplatin absorption were assessed by inductively coupled plasma mass spectrometry (ICP-MS) through CTR1 over-expression and knock-down. Further, the DNA binding activity of SP1 on CTR1 gene promoter was determined by dual-luciferase assay and chromatin immunoprecipitation (ChIP) assay. The functional regulation of p53 on SP1 was studied by nucleocytoplasmic separation assay and electrophoretic mobility shift assay (EMSA). The interaction between p53 and SP1 was verified by Co-Immunoprecipitation assay.

**Results:**

Under cisplatin treatment, p53 knock-out promoted CTR1 expression and cisplatin uptake, while p53 overexpression inhibited CTR1 expression and cisplatin uptake. Moreover, p53 regulated CTR1 level not by binding to CTR1 promoter directly but by suppressing the nuclear translocation of transcription factor specificity protein 1 (SP1). It was verified that SP1 is directly bound with CTR1 promoter. SP1 overexpression stimulated CTR1 expression, and SP1 knock-down attenuated CTR1 expression.

**Conclusion:**

The p53 might function as a negative regulator in CTR1 mediated cisplatin absorption, and the p53-SP1-CTR1 axis is a target for cisplatin resistance.

## Introduction

Osteosarcoma (OS), the most frequent primary malignant bone tumor, has an annual incidence of 3~4/million and predominantly affects children and young adolescents (1). Multimodal chemotherapy combined with surgery increases the 5-year survival rate from <20% to >60% in localized OS (2). However, the prognosis of OS patients remains stagnant in the last four decades, and drug resistance is a critical factor for the treatment dilemma (3).

Cisplatin is the first-line chemotherapeutic agent for OS. It links with genomic DNA, resulting in DNA loss and cytotoxicity (4). However, only 30% of OS patients respond to cisplatin treatment (5). Cisplatin resistance is multifactorial, including reduced cisplatin absorption, enhanced cisplatin detoxication, increased repair of DNA damage, and so on (3). Therefore, it is imperative to identify the mechanism of cisplatin resistance to improve the therapeutic effect of patients with OS.

Recent studies have identified that copper transporter 1 (CTR1) is the main influx transporter of cisplatin (6). High expression of CTR1 is correlated with enhanced platinum accumulation and chemotherapy sensitivity in small cell lung cancer, bladder cancer, and ovarian carcinoma cells (7–9). Our previous work also shows that upregulation of CTR1 increases cisplatin uptake and improves cisplatin sensitivity in OS cells (10). In small cell lung cancer, the transcription of CTR1 gene is activated by transcription factor specificity protein 1 (SP1) (11). At post-transcriptional level, CTR1 can be repressed by polypyrimidine tract binding protein 1 (PTBP1), microRNA-98-5p and microRNA-130a (12–14). However, the potential upper regulatory mechanisms of CTR1 in OS cells have never been reported.

As a housekeeping protein, tumor suppressor p53 is a sequence-specific DNA-binding protein that regulates transcription, which controls cell proliferation, DNA repair, and cell death (15). The mutation of p53 frequently occurs in cancer cells. It is generally believed that the presence of mutant p53 predisposes cancer development, promotes the survival of cancer cells, and is associated with ineffective therapeutic responses and unfavorable prognoses (16). Nevertheless, increasing studies find that patients harboring mutant or null p53 respond better to cisplatin than those harboring wild-type p53 in multiple cancer types (17–20). In addition, Fan et al. reported that induced expression of p53 in OS cell line SaOS-2 caused cells to be less sensitive to cisplatin (21). These findings suggest that p53 may promote cisplatin resistance under some conditions.

This study aimed to detect the protein expression of CTR1 in OS cells after cisplatin intervention, explore the role of transcription factors p53 and SP1 in the regulation of CTR1 expression, and understand the mechanism of p53 on cisplatin absorption in OS cells.

## Materials and methods

### Cell culture

Human OS cell lines U2OS and 143B were obtained from American Type Culture Collection (ATCC, Manassas, VA, USA). U2OS and 143B cells were cultured in McCoy’s 5A (HyClone Laboratories of Thermo Scientific, Logan, UT, USA) and high-glucose Dulbecco’s modified Eagle’s medium (DMEM, HyClone Laboratories of Thermo Scientific, Logan, UT, USA), respectively, which were supplemented with 10% fetal bovine serum (FBS) (Gibco, USA), 1% penicillin-streptomycin (10,000 U/mL) (Gibco, USA) at 37°C in 5% CO_2_ atmosphere.

### Reagents and antibodies

Cisplatin with a purity greater than 95% was purchased from Sigma-Aldrich Chemical Co. (St. Louis, MO, USA). The control IgG was obtained from Beyotime Biotech Ltd. (Shanghai, China). Antibodies against Bcl-2, Bax, caspase-3, SP1, CTR1, and p53 were obtained from Abcam (UK).

### RNA extraction and gene expressing plasmid construction

Total RNA of U2OS and 143B cells were extracted using Trizol reagent (Invitrogen, USA), and reverse transcription (RT) was conducted using an M-MLV kit (Promega, USA) of 500 ng total RNA. The cDNA was utilized to amplify p53 and SP1 genes by PCR using Q5 high-fidelity DNA polymerase (NEB, USA). For packaging the p53 gene expressing Lentivirus, the p53 gene was cloned into the pLenti-EGFP lentivirus vector. For transient transfection, the SP1 gene was constructed into the pFLAG-CMV2 plasmid. The primers used for amplified p53 and SP1 were listed in **Table 1**.

**Table 1:** Primer and probe sequence.

Name	Forward primer Sequence 5'----> 3"	Reverse primer Sequence 5'----> 3"
p53-Full-length	ATGGAGGAGCCGCAGTCAGA	TCAGTCTGAGTCAGGCCCTTCT
SP1-Full-length	ATGAGCGACCAAGATCACTCCA	TCAGAAGCCATTGCCACTGATA
p53-qPCR	CAGCACATGACGGAGGTTGT	TCATCCAAATACTCCACACGC
SP1-qPCR	GCTACCCCTACCTCAAAGGAAC	TTGCTGGTTCTGTAAGTTGGGA
CTR1-qPCR	TCTCACCATCACCCAACCACTT	ATTGATCACCAAACCGGAAAAC
GAPDH-qPCR	AAGGTGAAGGTCGGAGTCAA	AATGAAGGGGTCATTGATGG
CTR1-ChIP-qPCR	GAAATCGGTGTCGTCCTCG	GACTCTGGGAATACCCAGCTTC
CTR1-Probe-WT	GTCCCCCGCACCGAACATTGCGTTGGCTTTCACCGGGTTG	
CTR1-Probe-Mut	GTCCCCCGCACCGAACATTTATTTGGCTTTCACCGGGTTG	

### Lentivirus packaging and cell infection

For packaging p53-overexpression lentivirus, 293T cells were separated into 10cm plates pre-coated with Poly-D-lysine (Sigma) with 80% cell density. Then, cells were transfected with auxiliary plasmid psPAX2 and pMD2G, expressing pLenti-EGFP-p53 plasmid. Cellular supernatant was collected 48h post-transfection and filtrated with a 0.45μm filter membrane. To enhance the efficiency of infection, the supernatant was centrifuged at 20000 rpm/min for 2h. The virus pellet was dissolved with PBS. The p53 over-expression 143B cell line was established by lentivirus-mediated virus infection with MOI=5. Infected cells that expressed GFP were separated with flow cytometry 72h post-infection, and the expression of p53 was determined by Western blot.

### Generation of p53 deficient cell line by CRISPR-cas9 system

The p53 gene knock-out assay was performed on cultured U2OS cells using CRISPR-cas9 gene editing technology. The guide RNA targeting the p53 gene was constructed of sgRNA duplex using the True-Guide Synthetic gRNAs (Thermo Fischer Scientific, Waltham, USA). For the second approach, the vector-mediated editing approaches were employed, and the ssDNA was cloned into a pGCS plasmid vector. Then, the cultured U2OS cells were transfected with Cas9 plus each sgRNAs plasmid using a Lipofectamine 3000 (Thermo Fisher Scientific, USA), and GFP-positive cells were separated by FACS. Half of harvested were cultured in a 6-cm dish. Cellular DNA was isolated from another half of the cells using a QIAQuick PCR purification kit (Qiagen, Germany). The specific locus cleavage site on DNA was generated by PCR amplification with specific primer sequences that covers the CRISPR-Cas9 cut site. Then, the nuclease assay was carried out to detect and validate the CRISPR-Cas9 specificity. Nuclease assay was conducted by GeneArt Genomic Cleavage Detection Kit (Thermo Fisher Scientific, USA) as per the manufacturer’s guidelines, and CRISPR-Cas9 cut specificity was checked by agarose gel electrophoresis.

### CCK-8 cell proliferation assay

Cell proliferation and cytotoxicity of cisplatin were analyzed *via* CCK8 assay (Dojindo Laboratories, Kumamoto, Japan) according to its protocol. Briefly, the p53 over-expression, knock-out cells with indicated control cells were seeded into 96-well plates at a density of 3000 cells/well, then incubated for 0, 1, 2, 3, and 4 days. At the end of incubation, 10 μL of CCK-8 reagent was added to each well for 3h. The absorbance was measured at 450 nm wavelength on an automatic ELISA plate reader. The experiments were repeated three times with triplicate wells for each sample.

### Cell apoptosis assay

U2OS and 143B cells with different p53 genotypes were collected for the apoptosis assay using the Annexin V-FITC cell apoptosis Kit (BD, USA) according to the manufacturer’s instructions. Cells were seeded into 6-well plates and adhered to for 24 h. When the confluence reached 70-80%, cells were treated with or without cisplatin for 24h. Subsequently, cells were washed with cold phosphate-buffered saline (PBS) twice and resuspended in a 200 μl binding buffer. Then, the cells were stained with 5 μl of FITC for 20min in the dark. Finally, cells were added with 5 μl PI reagent before detection on the CytoFLEX flow cytometer (Beckman Coulter, Brea, CA, USA).

### Measurement of the intracellular Pt accumulation and the content of Pt‐DNA adducts

The samples of cells and DNA were used to measure total intracellular accumulation or the content of Pt-DNA adducts by inductively coupled plasma mass spectrometry (ICP-MS). The total DNA of cells were extracted by Genomic DNA Extraction Kit (TIANGEN Biotechnologies, Beijing, China) to determine the content of Pt-DNA adducts. The analyses were performed by the Department of Toxicology, School of Public Health, Peking University Health Science Center. The detection values were standardized by protein concentrations determined by BCA Protein Assay Kit or DNA concentrations measured by a UV‐Vis Spectro- photometer (Thermo Fisher Scientific, Waltham, USA).

### Western blot (WB) analysis

Differently treated cells were collected and lysed by RIPA lysis buffer (Cwbiotech, Beijing, China), and protein concentration was measured by a BCA Protein Assay Kit (Thermo Fisher Scientific, Waltham, USA). Briefly, an equal amount of lysate protein was separated by SDS-PAGE (10% gel) and transferred to polyvinylidene fluoride (PVDF) microporous membranes. After being blocked with 5% bovine serum albumin, membranes were blocked and incubated with the primary and secondary antibodies of interest in PBST at dilutions recommended by the manufacturer. Finally, protein bands were visualized by chemiluminescence using an ECL kit (Millipore, USA) according to the manufacturer’s protocol. The final average pixel of the protein band was calculated by normalizing to the loading control protein GAPDH.

### Real-time qPCR

Cellular total RNA of different treatment groups was extracted by using the RNeasy mini kit (QIAGEN, Germany) according to the manufacturer’s instructions. RNA was quantified using the Nano-one (Thermo Fisher Scientific, Waltham, USA), and quality was assessed by gel electrophoresis. The cDNA was synthesized using a Reverse Transcription kit (QIAGEN, Germany) and used as templates for qPCR. The data were analyzed by 2^-ΔΔCt^ method, and GAPDH was used as the internal control. The qPCR primers were listed in the **Table 1**.

### Nucleocytoplasmic separation assay

The NE-PER Kit (Thermo Fisher Scientific, Waltham, USA) was used to separate nuclear and cytoplasmic fractions from p53-OE, p53-KO, and indicated control cells. After digestion, the cells were resuspended in DPBS and buffer A containing DTT, Protease Inhibitor, Phosphates Inhibitor I, and Phosphates Inhibitor II for 15 min and then homogenized. The cells were centrifuged for 15 min at 4°C for 400×g, and the cytoplasm was isolated from the supernatant. Using PBS, nuclear isolation buffer (buffer B containing DTT, Protease Inhibitor, Phosphates Inhibitor I, and Phosphates Inhibitor II), and 0.3 mL RNase-free H_2_O, the precipitate was suspended in a centrifuge tube and incubated for 20 min on ice. The granules were then centrifuged to obtain the desired nuclear fragments. Using Tubulin as the cytoplasmic control and Lamin A as the nuclear control, the level of p53 and SP1 were detected using western blot in the cytoplasm or nucleus.

### Dual-luciferase activity assay

The sequence of CTR1 promoter region containing SP1 binding site was amplificated from DNA originated from U2OS cell and cloned into the pGL3-promoter dual-luciferase reporter plasmid (Promega, USA) to construct CTR1 expression reporter clone pGL3-CTR1-promoter. The 293T cells were seeded into a 12-well plate at a confluence of 70% one day prior to transfection. Then the luciferase reporter gene plasmid, SP1 expression plasmid, and pRL-TK were transfected into cells using Lipofectamine 3000 (Invitrogen, Thermo Fisher Scientific, Waltham, USA). After transfection for 48 h, cells were lysis, and luciferase activity was detected using the Dual-Luciferase Reporter Assay Kit (Promega, USA). The relative luciferase activity was determined by calculating the ratio of firefly fluorescence to Renilla fluorescence.

### ChIP−quantitative PCR (qPCR) assay

Chromatin immunoprecipitation (ChIP) assay was performed using a Magnetic ChIP kit (Thermo Fisher Scientific, Waltham, USA). Briefly, 143B cells were crosslinked with 1% formaldehyde followed by inactivation by 0.125 M glycine. The cells were lysed and sonicated to shear DNA to fragments. To immunoprecipitate protein-chromatin complexes, supernatants were incubated using 5 μg anti-p53 antibody overnight and 5 μg normal rabbit IgG antibodies per immunoprecipitation reaction as control. Then incubated for 2h after adding 50 μl of protein A/G agarose beads. Ten percent of the diluted supernatants were saved as input for normalization. Several washing steps were followed by protein digestion using cell lysis buffer. RT-PCR was used to amplify DNA fragments using specific primers. Reverse crosslinking was carried out at 65°C. DNA was subsequently purified. Immunoprecipitated chromatin was subjected to real-time qPCR using SYBR Green PCR Master Mix (Qiagen, Hilden, Germany). ChIP-qPCR enrichment analysis was performed, and each sample was normalized to the input, and the fold difference between the sample and IgG was calculated using 2^(−ΔΔCt^). The ChIP-qPCR primers were listed in **Table 1**.

### Electrophoretic mobility shift assay (EMSA)

DNA binding activity of the synthesized oligonucleotides to SP1 proteins was tested using LightShift Chemiluminescent EMSA Kit (Pierce, Thermo Scientific, USA). The 5’ biotin-labeled oligonucleotides were synthesized and labeled. Unlabeled oligonucleotides were included in the binding reaction for competition assays. U2OS cells with increasing amounts of CTR1 were incubated with probe DNA (50 nM) in a DNA binding buffer containing 20mM Tris-HCl, 50 mM NaCl, and 2mM DTT for 5 min. Then, the reaction mixtures were loaded on 5% polyacrylamide gels, and electrophoresis was performed in 1×TBE buffer at 100 V for 1.5 h on ice. After running the electrophoresis, the PAGE gel was photographed under UV light using chemiDoc™ XRS (Biorad, USA). Stains dye was used to stain the gel and photographed under a normal white light digital camera. The sequences of the oligonucleotides are listed in **Table 1**.

### Co-immunoprecipitation

U2OS cells were collected by trypsinization and washed with pre-cold PBS three times. Then cell pellet was lysed in RIPA lysis buffer (50 mM Tris-HCl (pH 7.4), 150 mM NaCl, 1% (vol/vol) NP-40, 1 mM EDTA, and 5% (vol/vol) glycerol) containing protease inhibitor cocktails. After 30 min, the lysed samples were centrifuged for 10 min at 4°C. A part of the lysates was saved as control. For immunoprecipitation, the rest of the lysates were incubated with the p53 antibody at 4°C overnight and then incubated with protein G agarose for 2h. The beads were washed 5 times with RIPA washing buffer (50 mM Tris–HCl, pH 7.4), 300 mM NaCl, 1% NP-40, 1 mM EDTA, and 5% glycerol) and then reconstituted in 50 μl 2×SDS loading buffer. All targeted protein bands were immunoblotted with the indicated antibodies.

### Statistical analysis

All experiments were repeated three times. All data were analyzed by SPSS Statistics 20.0 software and expressed as mean ± standard deviation (SD). Statistical analyses were carried out using the Student's t test or ANOVA followed by a Dunnett t test. Differences were considered statistically significant at a p <0.05.

## Results

### p53 attenuates the cytotoxic effect of cisplatin and inhibits cisplatin uptake in OS cells

**Figure 1:**
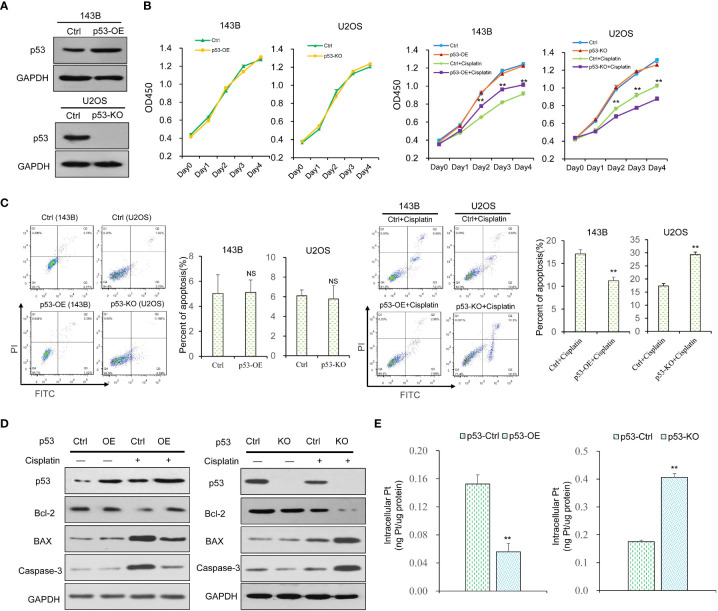
The effect of p53 on the viability, proliferation, apoptosis and cisplatin uptake in OS cells. **(A)** The p53 over-expression (p53-OE) 143B OS cell line was established by lentivirus-mediated infection, and p53 knock-out (p53-KO) U2OS OS cell line was built by CRISPR-cas9 gene editing technology. The expression of p53 was examined by western blot. GAPDH was used as the internal control. **(B)** Cell viability of p53-OE 143B cells and p53-KO U2OS cells treated with or without cisplatin (10 μg/ml) was determined by CCK8 assay. Data are representative of three independent experiments. **(C)** Cell apoptosis rate was detected by flow cytometry analysis using Annexin V and PI staining in p53-OE 143B cells and p53-KO U2OS cells treated with or without cisplatin (10 μg/ml). **(D)** The expression of apoptosis marker proteins in different p53-expressing OS cell lines with or without cisplatin incubation was determined by western blot. GAPDH was used as the internal control. **(E)** Cisplatin accumulation in p53-OE 143B cells and p53-KO U2OS cells treated with cisplatin (10 μg/ml) was detected by ICP-MS. Bars show mean ± SD. Ctrl, the control group; NS, no significance; **p < 0.01.

To explore the function of p53 on cisplatin, we built p53 over-expression (p53-OE) and p53 knock-out (p53-KO) OS cell lines (**Figure 1A**). CCK8 assays were used to examine the function of p53 on cell viability with or without cisplatin incubation. The results indicate that p53 alone has no significant impact on OS cell viability (**Figure 1B**). With cisplatin addition (10ug/mL), p53-OE cells were less sensitive to cisplatin compared with control cells, whereas p53-KO cells were more sensitive compared with control cells (**Figure 1B**). Cisplatin exerts cytotoxicity mainly through inducing cell apoptosis. Flow cytometry analysis showed that p53 expression has no significant impact on OS cell apoptosis without cisplatin addition (**Figure 1C**). Under cisplatin treatment, the cell apoptosis ratio was much lower in p53-OE group than in indicated control group, which was much higher in p53-KO group than indicated control group (**Figure 1C**). We next focused on whether apoptosis marker proteins of the classic apoptosis pathway could be associated with the outcome. All apoptosis marker proteins have no significant change without cisplatin treatment. However, the level of anti-apoptosis marker protein Bcl-2 increased upon cisplatin treatment in p53-OE cells. When p53 gene was knockout, Bcl-2 demonstrated reversed effect in the presence of cisplatin. The pro-apoptotic factor BAX protein and caspase-3 were also analyzed, confirming the protective effect of p53 on OS cells against cisplatin-induced cell death (**Figure 1D**). To explore whether the different effects of cell viability and apoptosis were due to the change of cisplatin uptake, inductively coupled plasma mass spectrometry (ICP-MS) was conducted to detect cisplatin accumulation in OS cells. The results confirmed a similar pattern with cell viability and apoptosis (**Figure 1E**). Taken together, these findings suggest that p53 exhibits a protective effect on cell viability and apoptosis in cisplatin treatment.

### CTR1 mediates cisplatin absorption and apoptosis in OS cells

**Figure 2:**
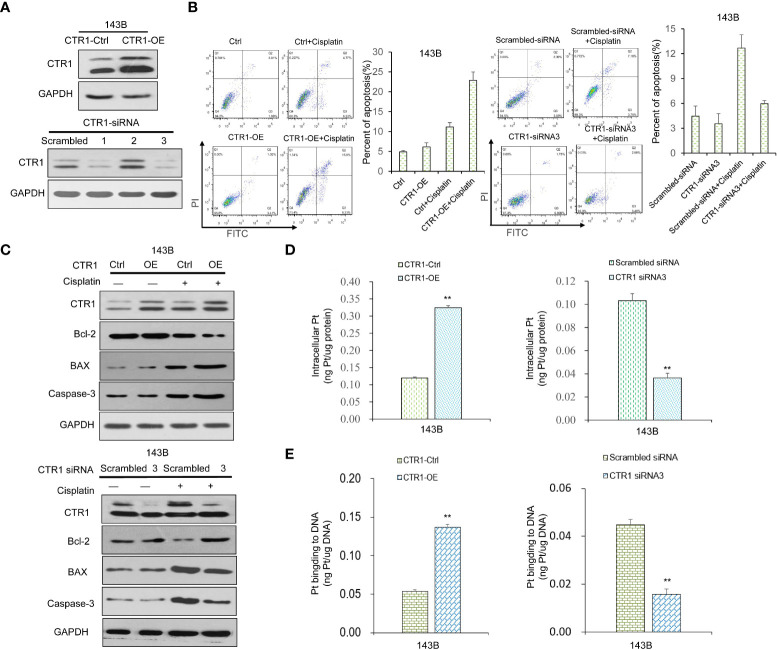
The function of CTR1 on apoptosis and cisplatin uptake in OS cells. **(A)** The 143B cell line was transfected with CTR1-overexpression (CTR1-OE) plasmid or indicated siRNAs to produce CTR1-OE or CTR1- knockdown (CTR1-KD) cells, respectively. The expression of CTR1 was examined by western blot. GAPDH was used as the internal control. **(B)** Cell apoptosis rate was measured in different CTR1-expressing 143B OS cell line with or without cisplatin incubation. Data are representative of three independent experiments. **(C)** The expression of apoptosis marker proteins in different CTR1-expressing 143B OS cell lines with or without cisplatin incubation was determined by western blot. GAPDH was used as the internal control. **(D)** Cisplatin accumulation and **(E)** content of Pt‐DNA adducts in CTR1-OE and CTR1-KD 143B cells treated with cisplatin (10 μg/ml) for 24h was detected by ICP-MS. Bars show mean ± SD. Ctrl, the control group; **p < 0.01.

To further explore the mechanism of the protective effect of p53 upon cisplatin treatment, we investigated whether CTR1 was involved. The CTR1 over-expression (CTR1-OE) and knock-down (CTR1-KD) OS cell lines were established by transfection of CTR1-OE plasmid and indicated siRNAs, respectively (**Figure 2A**). The effect of CTR1 level on cell apoptosis was determined. All groups showed a similar apoptosis ratio in the absence of cisplatin. Under cisplatin incubation, CTR1-OE cells had a significantly higher apoptosis ratio compared to control samples; while CTR1-KD cells showed a distinctly lower apoptosis ratio (**Figure 2B**). Similarly, the level of Bcl-2 decreased upon cisplatin treatment in CTR1-OE cells but increased in CTR1-KD cells, while BAX and caspase-3 increased in CTR1-OE cells but decreased in CTR1-KD cells (**Figure 2C**). To further confirm the cisplatin absorption mediated by CTR1, we performed ICP-MS on different CTR1-expressing cells treated with cisplatin. The ICP-MS provided similar results that there was a positive correlation between CTR1 protein expression level and cisplatin accumulation along with the content of Pt-DNA adducts (**Figures 2D, E**). These results indicated that the CTR1 level had a critical role in cisplatin absorption and cell apoptosis in OS cells.

### p53 inhibits CTR1 expression and cisplatin absorption in OS cells

**Figure 3:**
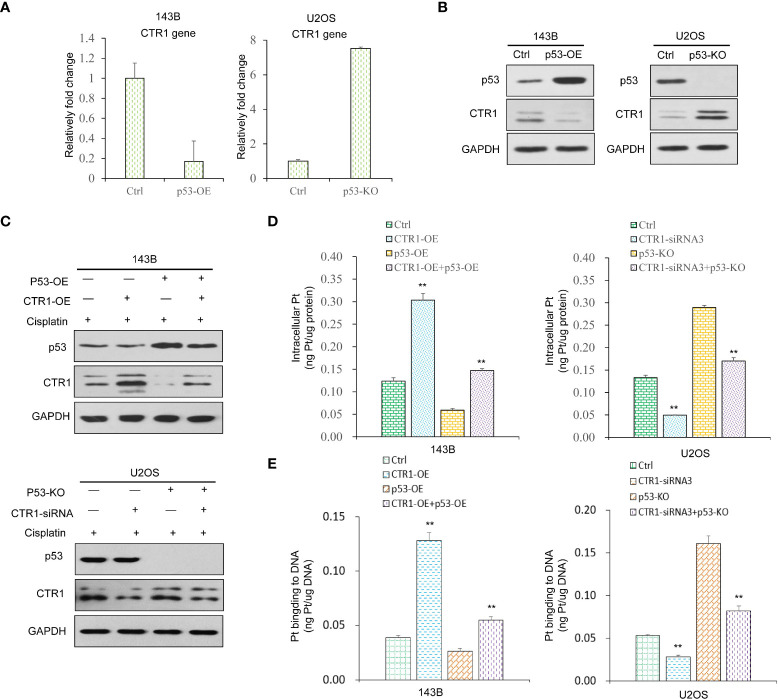
The function of p53 on CTR1 expression and cisplatin absorption in OS cells. **(A)** CTR1 mRNA expression was detected by RT-qPCR in p53 over-expression (p53-OE) 143B OS cell line and p53 knock-out (p53-KO) U2OS OS cell line. **(B)** CTR1 protein expression was detected by western blot in p53-OE 143B OS cell line and p53-KO U2OS OS cell lines. GAPDH was used as the internal control. **(C)** 143B cells overexpressed with the p53 gene and control cells were transfected with CTR1-OE plasmid upon cisplatin treatment. U2OS cells with p53 gene knock-out and control cells were transfected with CTR1-siRNA upon cisplatin treatment. The protein level of p53 and CTR1 was detected by western blot. GAPDH was used as the internal control. **(D)** Cisplatin accumulation **(E)** content of Pt‐DNA adducts in all groups (143B cells with p53-OE or CTR1-OE, U2OS cells with p53-KO or CTR1-siRNA) treated with cisplatin (10 μg/ml) for 24h was detected by ICP-MS. Data are representative of three independent experiments. Bars show mean ± SD. Ctrl, the control group; **p<0.01.

Considering that both p53 and CTR1 affected cisplatin uptake, we hypothesized a potential link between p53 and CTR1. We firstly conducted the RT-qPCR assays and found a significant decrease in CTR1 mRNA level upon p53 over-expression, while the p53-KO group upregulated CTR1 mRNA level (**Figure 3A**). Similarly, at the protein level, CTR1 was significantly upregulated in p53-KO cells compared to the control group, while downregulated in p53-OE cells (**Figure 3B**). These results demonstrated that the expression of CTR1 displayed a negative regulatory relationship with p53. Next, the regulation of p53 on CTR1 expression was explored by crossing cisplatin treatment in different p53 genotype cell lines. In the presence of cisplatin, the up-regulation of CTR1 was antagonized by p53 overexpression. Similarly, the down-regulation of CTR1 was saved by the knock-out of p53 under cisplatin treatment and CTR1 knock-down (**Figure 3C**). Furthermore, we investigated the effect of p53 on CTR1-mediated cisplatin absorption. ICP-MS showed the enhancement effect on cisplatin uptake and the content of Pt-DNA adducts by CTR1 overexpression was blocked by p53 overexpression. Likewise, the weakening effect on cisplatin uptake along with the content of Pt-DNA adducts by CTR1 knock-down was reversed by p53 knock-out (**Figures 3D, E**). These results indicate that p53 can negatively regulate CTR1 expression and subsequent cisplatin absorption in OS cells.

### The expression of CTR1 is not induced by p53-mediated direct promoter region binding

**Figure 4:**
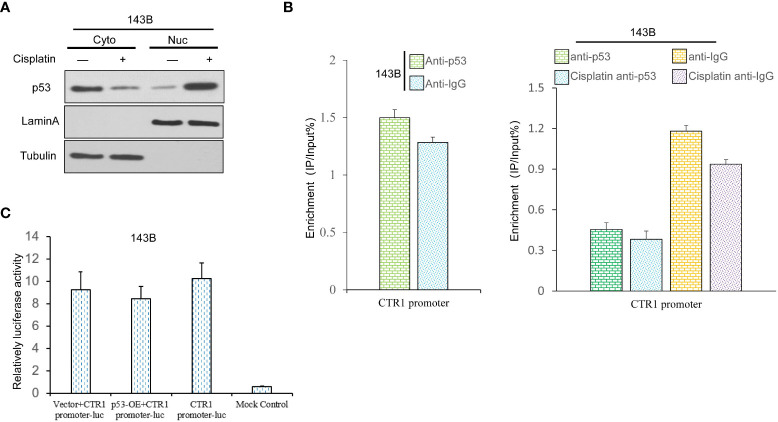
The p53 gene possesses a non-direct regulation effect on CTR1 promoter in OS cells. **(A)** The influence of cisplatin (10 μg/ml) on p53 nuclear translocation was determined by nucleocytoplasmic separation in 143B cells. The blotting of Lamin A and Tubulin were used as internal control to determine the separation effect. **(B)** Sheared chromatin from p53 expression cells treated with or without cisplatin was used to perform ChIP assays in 143B cells, and immunoprecipitated DNA was analyzed by qPCR using primers comprising the CTR1 promoter region. Results are expressed as a percentage of input DNA compared to IgG. **(C)** The CTR1 luciferase reporter assay was performed using p53-OE and control 143B cells. The data are presented as means of the ratio of firefly luciferase to renilla luciferase. Bars show mean ± SD.

The present results demonstrated that p53 inhibited cisplatin-induced expression of CTR1. It was hypothesized that p53 translocated from the cytoplasm to the nucleus to bind to the promoter of CTR1 gene in a cisplatin-induced manner. Therefore, U2OS cells were treated with or without cisplatin for 24 h, then cytoplasm and nuclear proteins were isolated to analyze the expression of p53. The nuclear located-p53 increased in cisplatin-treated cells, whereas the total p53 level had no apparent changes (**Figure 4A**). Next, the ChIP assay was conducted to determine the direct binding of p53 on the CTR1 promoter region. The amount of p53-bound DNA fragments of the CTR1 gene was measured as percentages to total input DNA subjected to immunoprecipitation followed by qPCR. Upon cisplatin treatment, the binding of p53 to the putative binding sites in the promoter elements of CTR1 showed no distinct enrichment over the input chromatin compared to that of an IgG-negative control antibody. Next, no chromatin enrichment was observed with primers from adjacent random regions (**Figure 4B**). We then assessed if U2OS cells transiently transfected with a CTR1-promoter luciferase reporter construct were activated by p53. The dual-luciferase activity demonstrated that p53 protein has no regulation effect on the luciferase activity of the CTR1-promoter, but only a slight decrease effect, indicating that p53 could not bind to CTR1-promoter directly (**Figure 4C**). These results suggest that p53 gene has no direct regulation on CTR1 gene expression.

### P53 mediates the expression of CTR1 through SP1 in OS cells

**Figure 5:**
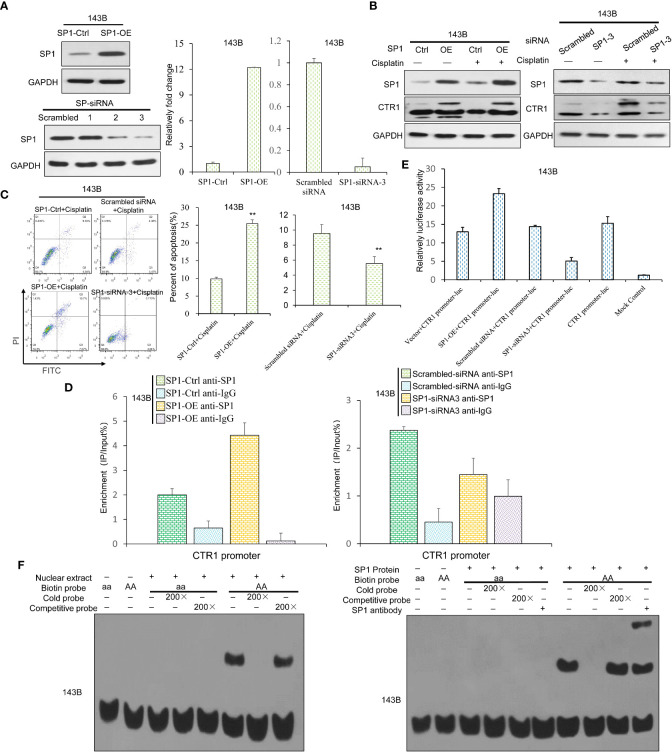
SP1 directly binds to CTR1 promoter and activates CTR1 transcription in OS cells. **(A)** The SP1 over-expression (SP1-OE) and knock-down (SP1-KD) 143B OS cell lines were established by transfection of SP1-OE plasmid or indicated siRNAs. SP1 mRNA and protein levels were confirmed by western blot and RT-qPCR. **(B)** The expression level of CTR1 in SP1-OE and SP1-KD 143B cells after cisplatin treatment were detected using western blot. **(C)** Cell apoptosis rate was measured in different SP1-expressing 143B cells with cisplatin incubation. Data are representative of three independent experiments. **(D)** Chromatin immunoprecipitation assay was performed, and the binding ability of SP1 protein to the CTR1 promoter was measured with ChIP-qPCR. **(E)** The binding ability of SP1 was detected *via* luciferase activity upon cisplatin stimulation. The pGL3-Basic vector showed no activity when stimulated with cisplatin. **(F)** The binding activity of SP1 on the CTR1 promoter was determined by EMSA assay in 143B cells. The activity of the wild-type CTR1 promoter and the mutant was tested with an EMSA assay. Bars show mean ± SD. Ctrl, the control group; **p < 0.01.

Transcription factor SP1 is reported to activate CTR1 expression (11). We constructed SP1-OE plasmid and siRNA for SP1 knock-down to explore the potential link between SP1 and CTR1 in OS cells. The over-expression and knock-down of SP1 were confirmed by western blot and RT-qPCR (**Figure 5A**). SP1 overexpression stimulated CTR1 expression in a cisplatin-independent manner, and SP1 knock-down attenuated CTR1 expression (**Figure 5B**). Likewise, SP1 overexpression group had a higher rate of cell apoptosis than the control group, whereas SP1 knock-down nearly completely abolished the apoptosis-inducing effect of cisplatin on U2OS cells (**Figure 5C**). The next objective was to determine whether the regulation of SP1 on CTR1 gene through a direct manner. It was shown that SP1 responded strongly to cisplatin treatment on the CTR1 promoter upon SP1-OE plasmid compared with the control vector. In contrast, SP1 knock-down had consistent low binding ability on CTR1 promoter compared with the scrambled siRNA upon cisplatin treatment (**Figure 5D**). Furthermore, the dual-luciferase analysis showed that the luciferase activity increased after co-transfection of SP1-OE plasmid compared with vector control, and knockdown of SP1 by gene-specific siRNA3 significantly decreased luciferase activity by more than 80%. PGL3-Basic luciferase vector that lacks CTR1 promoter domain did not demonstrate a change in activity (**Figure 5E**). Finally, the EMSA assay further confirmed that recombinant SP1 protein could directly bind to the sequences with high affinity in the CTR1 promoter. When the binding sequences were mutated, SP1 had no binding capacity (**Figure 5F**). Thus, the data suggest that SP1 protein can bind to the CTR1 promoter directly, and SP1 is a potent stimulation reagent on CTR1 expression.

### P53 inhibits SP1 nuclear transfer and mediates the expression of CTR1 by direct binding to SP1

**Figure 6:**
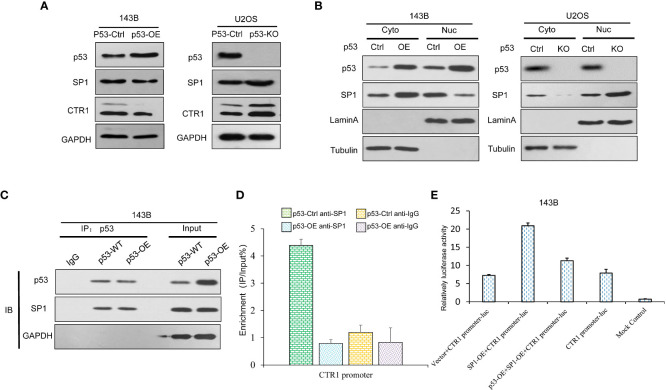
p53 suppresses CTR1 transcription by directly binding with SP1 in OS cells. **(A)** The expression of SP1 was detected by western blot in p53 over-expression (p53-OE) 143B OS cell line and p53 knock-out (p53-KO) U2OS OS cell line. GAPDH was used as the internal control. **(B)** Nuclear-cytoplasmic separation was utilized to investigate SP1 nuclear translocation in different p53 expression cell lines. The blotting of Lamin A and Tubulin were used as internal control to determine the separation effect. **(C)** The interaction between p53 and SP1 was determined by Co-IP in 143B cells. Proteins precipitated using anti-Flag antibodies were resolved by SDS-PAGE. **(D)** The impact of p53 on SP1 binding ability in CTR1 promoter was determined by ChIP assay in different p53-expressing 143B cell lines. **(E)** The dual-luciferase activity assay was utilized to confirm the influence of p53 on SP1 binding ability in 143B cells. Bars show mean ± SD.

The inhibition of p53 on cisplatin absorption by CTR1 has been demonstrated above. SP1 could enhance CTR1 expression and promotes cisplatin-induced cell apoptosis. We detected SP1 protein levels in p53-OE and KO cell lines. The result demonstrated no significant change of SP1 in different p53 expression cell lines (**Figure 6A**). Given the SP1 level above, we further explore whether the SP1 function of CTR1 is partially through nuclear transfer. Therefore, we examined the effect of SP1 nuclear transfer with or without p53 protein. As shown in **Figure 6B**, the nuclear-cytoplasmic separation assay showed that over-expression of p53 inhibited SP1 protein nuclear transfer. Consistently, depleting p53 induced a remarkable increase in the amount of SP1 in the nucleus. Thus, we identified that p53 could mediate SP1 nuclear transfer. To further explore the nuclear transfer of p53, we focused on the protein interacting with CTR1. We immunoprecipitated p53 protein with antibody and identified that p53 directly interacted with SP1 (**Figure 6C**). Based on the results of nuclear-cytoplasmic separation and CoIP assay, we hypothesized that p53 might hinder the content of nuclear SP1, which bind to the CTR1 promoter. The expression of SP1 and CTR1 promoter complex was analyzed by ChIP assay to confirm this. The result demonstrated a significant increase in p53-KO cells expression of the SP1-CTR1 promoter complex, which was downregulated by p53 over-expression (**Figure 6D**). To validate the regulation of p53 protein on SP1 mediated CTR1 expression, a luciferase assay was carried out. As shown in **Figure 6E**, the luciferase promoter activity was increased with SP1-overexpression, while p53-overexpression effectively antagonized the enhancement of luciferase activity on CTR1 promoter mediated by SP-1 overexpression. These results suggest that activated p53 impeded the nuclear translocation traffics of SP1 to the nucleus and subsequent binding to CTR1 sites in the promoter region. Taken together, p53 may function through downregulating CTR1, which was involved in the SP1-mediated nuclear translocation.

## Discussion

The prognosis of OS has reached a plateau nowadays; even immune therapy like PD1 inhibitors also do not show encouraging activity (22). Increasing evidence has shed light on the potential value of drug transporter CTR1 in OS treatment (23). This study investigated the association between p53 and cisplatin absorption in OS cells. Our results have provided mechanistic insights into the process of CTR1-mediated cisplatin absorption by uncovering the critical role of p53 and SP1 in CTR1 regulation. It is a novel mechanism relevant to p53-driven drug uptake and cancer development.

Tumor suppressor p53 is widely known for its vital role in inducing cell cycle arrest, apoptosis, and senescence. One hallmark of cancer is the altered metabolic activity of cancer cells, and recent studies have revealed that p53 also regulates cell metabolism (24). In 2004, Schwartzenberg-Bar-Yoseph et al. firstly reported that p53 repressed glucose transporter 1 (GLUT1) and GLUT4 gene transcription to decrease glucose uptake in human OS cells, human embryonal rhabdomyosarcoma cells, and murine myoblasts (25). Except for transcriptional suppression, p53 also inhibits IKKβ/NF-κB signaling pathway to downregulate GLUT1 and GLUT4 expression indirectly (26). Cancer cells mainly rely on glycolysis as the predominant source of ATP, even in the presence of oxygen, known as the Warburg effect (27). Studies have shown that p53 suppresses the expression or the activity of multiple enzymes to inhibit glycolysis, including hexokinase II (HK-II) (28), phosphofructokinase-1 (PFK-1) (29), and phosphoglycerate kinase-1 (PGK-1) (30). In addition, p53 also regulates lipid metabolism, mitochondrial activities (25), and the synthesis of serine (31). Jiang et al. found that p53 repressed the expression of SLC7A11, a key component of the cystine/glutamate antiporter, to inhibit cystine uptake and sensitize tumor cells to ferroptosis (32). However, whether p53 regulates metal ion transporter to affect metal element absorption and metabolism has not been reported. In this study, we found that p53 repressed transporter CTR1 expression to inhibit cisplatin uptake and sensitize OS cells to cisplatin treatment. Different from the above studies show that p53 regulates the expression of the transporters by direct transcription suppression, our study indicates that p53 mediates CTR1 expression by affecting the translocation of SP1. The potential reason for the difference may be that glucose metabolism determines cell death and survival all the time, while drug uptake only occurs when cancer cells face drug stress. Seen from this point, p53 may be deemed as a “bad protein”, who builds a barrier between cisplatin and OS cells. The finding proposes p53 is a crucial factor in regulating cisplatin uptake, which supplies a novel perspective to understanding the role of p53 in cisplatin resistance and as a potential prognostic biomarker before cisplatin treatment.

The human P53 gene comprises 13 exons which generate 12 different p53 isoforms protein through combination usage of alternative splicing and distinct promoters located in the upstream of exon 1 (P1) and within intron 4 (P2). The role of various p53 isoforms display different effects on cell fate and function by modulating p53 activity. Furthermore, the abnormally expressed TP53 mutations has been extensively documented in many tumors (33, 34). In this study, the knockout site is located on the third exon of p53 gene resulting in frameshift mutation and early termination of protein translation. Our strategy disturbed the function of full length of p53 protein, however, the isoform of p53 utilized the P2 promoter for transcription initiation may not be destroyed. A study of P2-driven expression of the mutant R167H-Δ152p53 isoform determine that Δ152p53α isoform play a critical role in the malignant transformation and tumor spectrum in pigs. The higher expression of Δ152p53α isoform was observed in pigs with early onset of OS (35). Yet, potentially function of different TP53 isoforms still remain unclear in CTR1-mediated cisplatin absorption in the OS. Further research is required to prove the existence of other isoforms of p53 and the potential functions of them in OS cellular lines.

Like p53, SP1 is also viewed as a housekeeping gene implicated in various essential biological processes including cell growth, differentiation, apoptosis, and carcinogenesis (36). Similar to p53, SP1 regulates glycolysis by transactivating the GLUT1 promoter (37). SP1 induces the transcription of cellular genes that contain CG-rich-binding sites in their promoters (36). Besides transporting platinum, CTR1 is firstly found to transport copper. Kuo’s team reported that both zinc-finger and glutamine-rich domains of SP1 were sensors of the copper, which enabled SP1 to regulate CTR1 expression under copper stress (11, 38). Consistent with Kuo’s findings, we find that SP1 activates CTR1 transcription. However, whether cisplatin may be the potential stress to stimulate SP1 needs further exploration.

It has been a consensus that CTR1 is the pivotal influx-transporter of platinum; however, few studies have focused on the regulatory mechanism of the CTR1 in cisplatin uptake. Except for SP1, Porcu et al. report that the transcription factor c-myc binds the transcription start site of CTR1 promoter region to regulate CTR1 expression in hepatocellular carcinoma cells (39). The other studies all find post-transcriptional regulatory mechanisms, including polypyrimidine tract binding protein 1 (PTBP1), microRNA-98-5p, microRNA-130a and microRNA-21 (12–14, 40). Our study confirms the transcriptional activation function of SP1 in OS cells. What is different is that our work demonstrates that p53 works at the upper stream of SP1 in the regulation of cisplatin uptake, which constitutes the p53-SP1-CTR1 axis. Similar to our finding, some studies have highlighted that SP1 and p53 often cooperate to regulate many tumor-related signaling pathways (41). In addition to transport copper and platinum, a recent study shows that CTR1 is rapidly sulfenylated at Cys189 at its cytosolic C terminus after stimulation with vascular endothelial growth factor (VEGF), which induces CTR1-vascular endothelial growth factor receptor type 2(VEGFR2) disulfide bond formation and their co-internalization to early endosomes, driving sustained VEGFR2 signaling to enhance angiogenesis (42). It is needed to explore further whether cytokines, like VEGF, have a possible function in the process of CTR1-mediated cisplatin absorption.

At present, drug resistance has been seriously limited the treatment effect of chemotherapy on tumor. The pharmacokinetic (PK) study provide a scientific basis for the safe and effective use of therapeutic antitumor drugs. So, the efficacy of drug administration and efflux is a determinant of PK which consistent with drug resistance (43). The efficacy of anti-tumor drug absorption depends on characteristics of drug and cell type. Previous studies have shown that the influx of cisplatin is due to passive diffusion across the cell membrane. Modern studies reveal that CTR1 also proposed a pivotal role in the uptake of cisplatin (4, 44). However, the mode of passive transport of cisplatin in osteosarcoma cell lines has been poorly studied. In our study, knockdown of CTR1 gene significantly decreased cisplatin intake, suggesting that CTR1-mediated cisplatin intake is one of the main modes of intracellular cisplatin absorption. However, the contribution of passive transport to cisplatin intake should also be considered. Many studies of ultrasound displayed the antagonistic effect to drug resistance through the enhancement of intracellular anticancer drug level (43, 45). The exists of regulatory effect of p53 on CTR1 is unknown in the ultrasound-mediated anti-tumor effects. These antitumor therapies that increase intracellular cisplatin content combined with p53 genotype analysis should be conducted in subsequent studies and need further research.

In conclusion, this study demonstrates that p53 inhibits cisplatin uptake in OS cells. Further mechanism studies indicate that inhibitory effect results from p53-mediated suppression of SP1 nuclear transfer and subsequent downregulation of CTR1. However, insufficient *in vivo* data has been acquired in this study to determine the regulation effect of cisplatin absorption by p53. We will cover these aspects of study in future with *in vivo* tumor model system and clinical osteosarcoma samples. To our knowledge, the current study is the first to demonstrate that p53-dependent SP1 activity regulates cisplatin absorption, which provides evidence for a new oncogenic role of p53 in OS. Thus, the multiple p53 genotypes important for cisplatin transport may be a promising direction for personal patient treatment in OS.

## Data availability statement

The original contributions presented in the study are included in the article/**Supplementary Material**. Further inquiries can be directed to the corresponding author.

## Author contributions

LY, YS, and H-LW provided key design of these studies and performed the experiments. Q-YD, JG, and L-SH analyzed the data. W-HW, Z-PG, and LY wrote the manuscript. B-SY conceived the project and supervised the study. All authors read and approved the final manuscript.

## References

[1] Casali PG Bielack S Abecassis N Aro HT Bauer S Biagini R. Bone sarcomas: ESMO-PaedCan-EURACAN clinical practice guidelines for diagnosis, treatment and follow-up. Ann Oncol (2018) 29(Supplement_4):iv79–95. doi: 10.1093/annonc/mdy310 30285218

[2] Smeland S Bielack SS Whelan J Bernstein M Hogendoorn P Krailo MD. Survival and prognosis with osteosarcoma: Outcomes in more than 2000 patients in the EURAMOS-1 (European and American osteosarcoma study) cohort. Eur J Cancer (2019) 109:36–50. doi: 10.1016/j.ejca.2018.11.027 30685685 PMC6506906

[3] Lilienthal I Herold N. Targeting molecular mechanisms underlying treatment efficacy and resistance in osteosarcoma: A review of current and future strategies. Int J Mol Sci (2020) 21(18). doi: 10.3390/ijms21186885 PMC7555161 32961800

[4] Ghosh S. Cisplatin: The first metal based anticancer drug. Bioorg Chem (2019) 88:102925. doi: 10.1016/j.bioorg.2019.102925 31003078

[5] Chou AJ Gorlick R. Chemotherapy resistance in osteosarcoma: Current challenges and future directions. Expert Rev Anticancer Ther (2006) 6(7):1075–85. doi: 10.1586/14737140.6.7.1075 16831079

[6] Chen HH Chen WC Liang ZD Tsai WB Long Y Aiba I. Targeting drug transport mechanisms for improving platinum-based cancer chemotherapy. Expert Opin Ther Targets (2015) 19(10):1307–17. doi: 10.1517/14728222.2015.1043269 PMC4846984 26004625

[7] Kim ES Tang X Peterson DR Kilari D Chow CW Fujimoto J. Copper transporter CTR1 expression and tissue platinum concentration in non-small cell lung cancer. Lung Cancer (2014) 85(1):88–93. doi: 10.1016/j.lungcan.2014.04.005 24792335 PMC4090351

[8] Kilari D Iczkowski KA Pandya C Robin AJ Messing EM Guancial E. Copper transporter-CTR1 expression and pathological outcomes in platinum-treated muscle-invasive bladder cancer patients. Anticancer Res (2016) 36(2):495–501. 26851002

[9] Lee YY Choi CH Do IG Song SY Lee W Park HS. Prognostic value of the copper transporters, CTR1 and CTR2, in patients with ovarian carcinoma receiving platinum-based chemotherapy. Gynecol Oncol (2011) 122(2):361–5. doi: 10.1016/j.ygyno.2011.04.025 21570711

[10] Yong L Ma Y Liang C He G Zhao Z Yang C. Oleandrin sensitizes human osteosarcoma cells to cisplatin by preventing degradation of the copper transporter 1. Phytother Res (2019) 33(7):1837–50. doi: 10.1002/ptr.6373 31050072

[11] Song I-S Chen HHW Aiba I Hossain A Liang ZD Klomp LW J. Transcription factor Sp1 plays an important role in the regulation of copper homeostasis in mammalian cells. Mol Pharmacol (2008) 74(3):705–13. doi: 10.1124/mol.108.046771 PMC2574735 18483225

[12] Cheng C Ding Q Zhang Z Wang S Zhong B Huang X. PTBP1 modulates osteosarcoma chemoresistance to cisplatin by regulating the expression of the copper transporter SLC31A1. J Cell Mol Med (2020) 24(9):5274–89. doi: 10.1111/jcmm.15183 PMC7205786 32207235

[13] Jiang P Wu X Wang X Huang W Feng Q. NEAT1 upregulates EGCG-induced CTR1 to enhance cisplatin sensitivity in lung cancer cells. Oncotarget (2016). doi: 10.18632/oncotarget.9712 PMC5190027 27270317

[14] Feng C Ma F Hu C Ma J A Wang J Zhang Y. SOX9/miR-130a/CTR1 axis modulates DDP-resistance of cervical cancer cell. Cell Cycle (2018) 17(4):448–58. doi: 10.1080/15384101.2017.139.5533. PMC5927693 29099271

[15] Kastenhuber ER Lowe SW. Putting p53 in context. Cell (2017) 170(6):1062–78. doi: 10.1016/j.cell.2017.08.028 PMC5743327 28886379

[16] Sabapathy K Lane DP. Therapeutic targeting of p53: all mutants are equal, but some mutants are more equal than others. Nat Rev Clin Oncol (2018) 15(1):13–30. doi: 10.1038/nrclinonc.2017.151 28948977

[17] Lavarino C Pilotti S Oggionni M Gatti L Perego P Bresciani G. p53 gene status and response to platinum/paclitaxel-based chemotherapy in advanced ovarian carcinoma. J Clin Oncol (2000) 18(23):3936–45. doi: 10.1200/JCO.2000.18.23.3936 11099323

[18] Choi W Porten S Kim S Willis D Plimack ER Hoffman-Censits J. Identification of distinct basal and luminal subtypes of muscle-invasive bladder cancer with different sensitivities to frontline chemotherapy. Cancer Cell (2014) 25(2):152–65. doi: 10.1016/j.ccr.2014.01.009 PMC4011497 24525232

[19] Mcconkey DJ Choi W Shen Y Lee IL Porten S Matin SF. A prognostic gene expression signature in the molecular classification of chemotherapy-naive urothelial cancer is predictive of clinical outcomes from neoadjuvant chemotherapy: A phase 2 trial of dose-dense methotrexate, vinblastine, doxorubicin, and cisplatin with bevacizumab in urothelial cancer. Eur Urol (2016) 69(5):855–62. doi: 10.1016/j.eururo.2015.08.034 PMC4775435 26343003

[20] Silver DP Richardson AL Eklund AC Wang ZC Szallasi Z Li Q. Efficacy of neoadjuvant cisplatin in triple-negative breast cancer. J Clin Oncol (2010) 28(7):1145–53. doi: 10.1200/JCO.2009.22.4725 PMC2834466 20100965

[21] Fan J Bertino JR. Modulation of cisplatinum cytotoxicity by p53: effect of p53-mediated apoptosis and DNA repair. Mol Pharmacol (1999) 56(5):966–72. doi: 10.1124/mol.56.5.966 10531402

[22] Tawbi HA Burgess M Bolejack V Van Tine BA Schuetze SM Hu J. Pembrolizumab in advanced soft-tissue sarcoma and bone sarcoma (SARC028): a multicentre, two-cohort, single-arm, open-label, phase 2 trial. Lancet Oncol (2017) 18(11):1493–501. doi: 10.1016/S1470-2045(17)30624-1 PMC7939029 28988646

[23] Serra M Hattinger CM. The pharmacogenomics of osteosarcoma. Pharmacogenomics J (2017) 17(1):11–20. doi: 10.1038/tpj.2016.45 27241064

[24] Napoli M Flores ER. The p53 family orchestrates the regulation of metabolism: physiological regulation and implications for cancer therapy. Br J Cancer (2017) 116(2):149–55. doi: 10.1038/bjc.2016.384 PMC5243983 27884017

[25] Schwartzenberg-Bar-Yoseph F Armoni M Karnieli E. The tumor suppressor p53 down-regulates glucose transporters GLUT1 and GLUT4 gene expression. Cancer Res (2004) 64(7):2627–33. doi: 10.1158/0008-5472.CAN-03-0846 15059920

[26] Xi Y Zhang Y Pan J Chen S Lu S Shen F. Triptolide dysregulates glucose uptake *via* inhibition of IKKbeta-NF-kappaB pathway by p53 activation in cardiomyocytes. Toxicol Lett (2020) 318:1–11. doi: 10.1016/j.toxlet.2019.10.001 31618665

[27] Pavlova NN Zhu J Thompson CB. The hallmarks of cancer metabolism: Still emerging. Cell Metab (2022) 34(3):355–77. doi: 10.1016/j.cmet.2022.01.007 PMC8891094 35123658

[28] Mathupala SP Heese C Pedersen PL. Glucose catabolism in cancer cells. the type II hexokinase promoter contains functionally active response elements for the tumor suppressor p53. J Biol Chem (1997) 272(36):22776–80. doi: 10.1074/jbc.272.36.22776 9278438

[29] Bensaad K Tsuruta A Selak MA Vidal MN Nakano K Bartrons R. TIGAR, a p53-inducible regulator of glycolysis and apoptosis. Cell (2006) 126(1):107–20. doi: 10.1016/j.cell.2006.05.036 16839880

[30] Luo X Ge J Chen T Liu J Liu Z Bi C. LHX9, a p53-binding protein, inhibits the progression of glioma by suppressing glycolysis. Aging (Albany NY) (2021) 13(18):22109–19. doi: 10.18632/aging.203436 PMC8507291 34536269

[31] Hitosugi T Zhou L Elf S Fan J Kang HB Seo JH. Phosphoglycerate mutase 1 coordinates glycolysis and biosynthesis to promote tumor growth. Cancer Cell (2012) 22(5):585–600. doi: 10.1016/j.ccr.2012.09.020 23153533 PMC3500524

[32] Jiang L Kon N Li T Wang S J Su T Hibshoosh H. Ferroptosis as a p53-mediated activity during tumour suppression. Nature (2015) 520(7545):57–62. doi: 10.1038/nature14344 25799988 PMC4455927

[33] Hayman L Chaudhry WR Revin VV. What is the potential of p53 isoforms as a predictive biomarker in the treatment of cancer? Expert Rev Mol Diagn (2019) 19(2):149–59. doi: 10.1080/14737159.2019.1563484 30582376

[34] Anbarasan T Bourdon JC. The emerging landscape of p53 isoforms in physiology, cancer and degenerative diseases. Int J Mol Sci (2019) 20(24):6257. doi: 10.3390/ijms20246257 31835844 PMC6941119

[35] Niu G Hellmuth I Flisikowska T Pausch H Rieblinger B Carrapeiro A. Porcine model elucidates function of p53 isoform in carcinogenesis and reveals novel circTP53 RNA. Oncogene (2021) 40(10):1896–908. doi: 10.1038/s41388-021-01686-9 PMC7946636 33603167

[36] Vizcaino C Mansilla S Portugal J. Sp1 transcription factor: A long-standing target in cancer chemotherapy. Pharmacol Ther (2015) 152:111–24. doi: 10.1016/j.pharmthera.2015.05.008 25960131

[37] Yin J Shi Z Wei W Lu C Wei Y Yan W. MiR-181b suppress glioblastoma multiforme growth through inhibition of SP1-mediated glucose metabolism. Cancer Cell Int (2020) 20:69. doi: 10.1186/s12935-020-1149-7 32158359 PMC7057587

[38] Liang ZD Tsai WB Lee MY Savaraj N Kuo MT. Specificity protein 1 (sp1) oscillation is involved in copper homeostasis maintenance by regulating human high-affinity copper transporter 1 expression. Mol Pharmacol (2012) 81(3):455–64. doi: 10.1124/mol.111.076422 PMC3286298 22172574

[39] Porcu C Antonucci L Barbaro B Illi B Nasi S Martini M. Copper/MYC/CTR1 interplay: a dangerous relationship in hepatocellular carcinoma. Oncotarget (2018) 9(10):9325–43. doi: 10.18632/oncotarget.24282 PMC5823635 29507693

[40] Gaudelot K Gibier JB Pottier N Hemon B Van Seuningen I Glowacki F. Targeting miR-21 decreases expression of multi-drug resistant genes and promotes chemosensitivity of renal carcinoma. Tumour Biol (2017) 39(7):1010428317707372. doi: 10.1177/1010428317707372 28714373

[41] Beishline K Azizkhan-Clifford J. Sp1 and the ‘hallmarks of cancer’. FEBS J (2015) 282(2):224–58. doi: 10.1111/febs.13148 25393971

[42] Das A Ash D Fouda AY Sudhahar V Kim YM Hou Y. Cysteine oxidation of copper transporter CTR1 drives VEGFR2 signalling and angiogenesis. Nat Cell Biol (2022) 24(1):35–50. doi: 10.1038/s41556-021-00822-7 35027734 PMC8851982

[43] Zhang Y Li JY Yu TH. Pharmacokinetic profiles of cancer sonochemotherapy. Expert Opin Drug Delivery (2017) 14(6):745–53. doi: 10.1080/17425247.2016.1232248 27589927

[44] Arnesano F Natile G. Interference between copper transport systems and platinum drugs. Semin Cancer Biol (2021) 76:173–88. doi: 10.1016/j.semcancer.2021.05.023 34058339

[45] Yu TH Luo L Wang L. Ultrasound as a cancer chemotherapy sensitizer: the gap between laboratory and bedside. Expert Opin Drug Deliv (2016) 13(1):37–47. doi: 10.1517/17425247.2015.1083008 26328944

