# Supplementary material for: p53 inhibits CTR1-mediated cisplatin absorption by suppressing SP1 nuclear translocation in osteosarcoma
Source: Front Oncol. 2023 Jan 26;12:1047194. doi: 10.3389/fonc.2022.1047194 (PMC9910081; doi:10.3389/fonc.2022.1047194)

Figure. 1A

p53

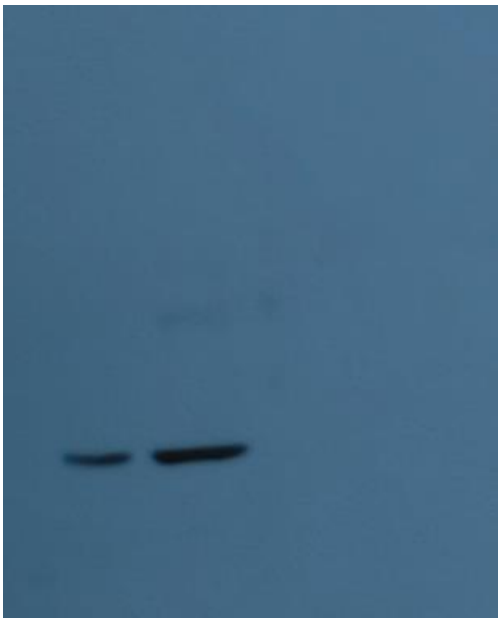

GAPDH

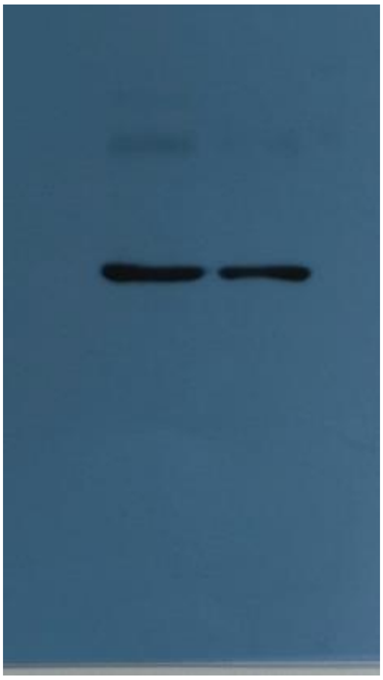

p53

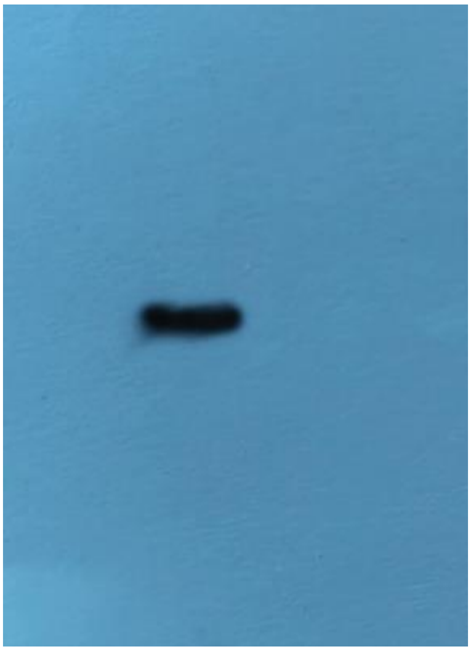

GAPDH

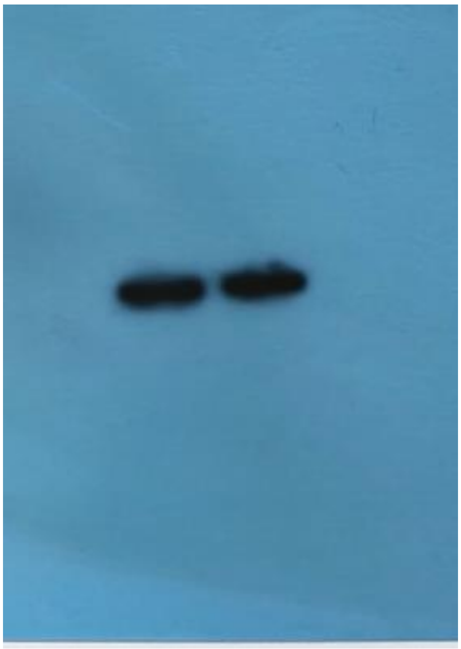

Figure. 1D（左图）

p53

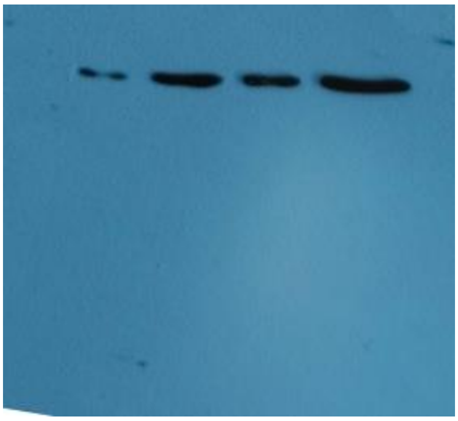

Bcl-2

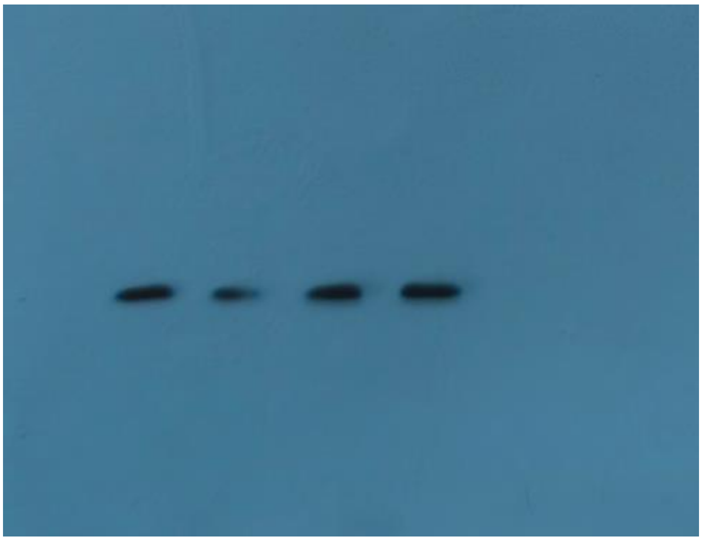

Bax

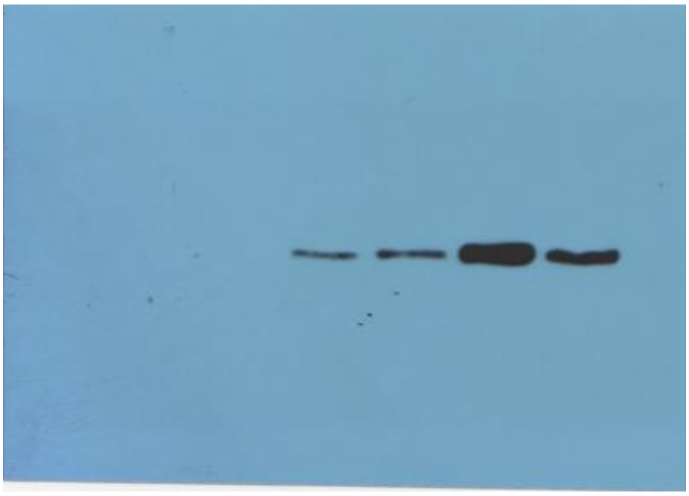

Caspase-3

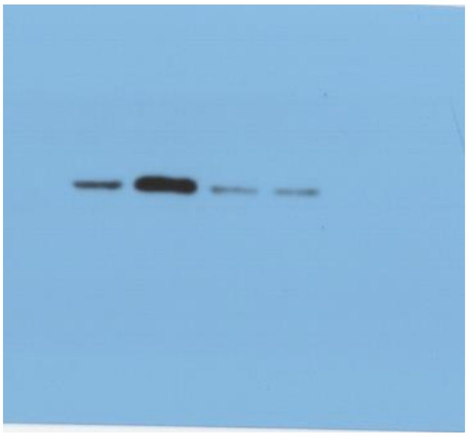

GAPDH

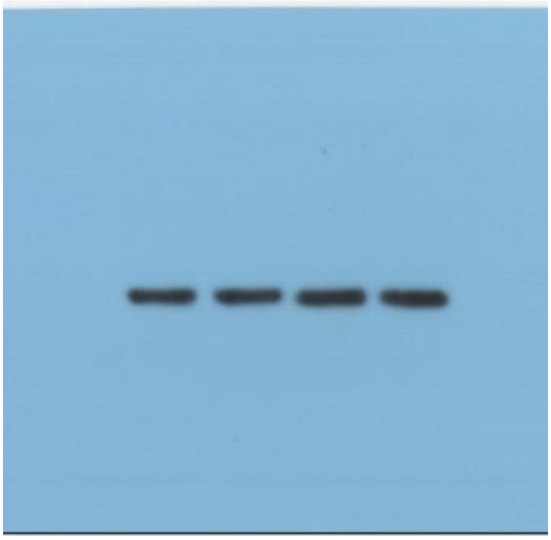

Figure. 1D（右图）

p53

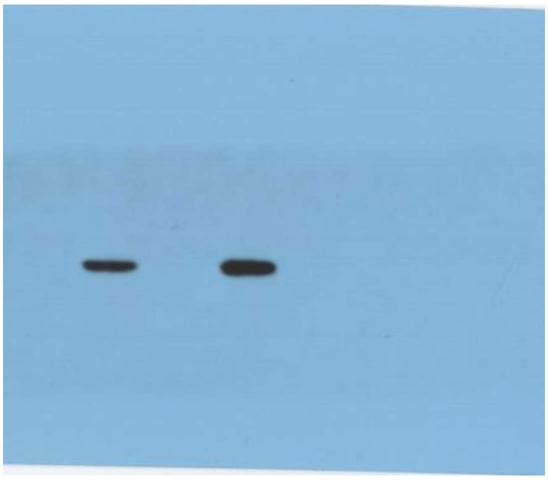

Bcl2

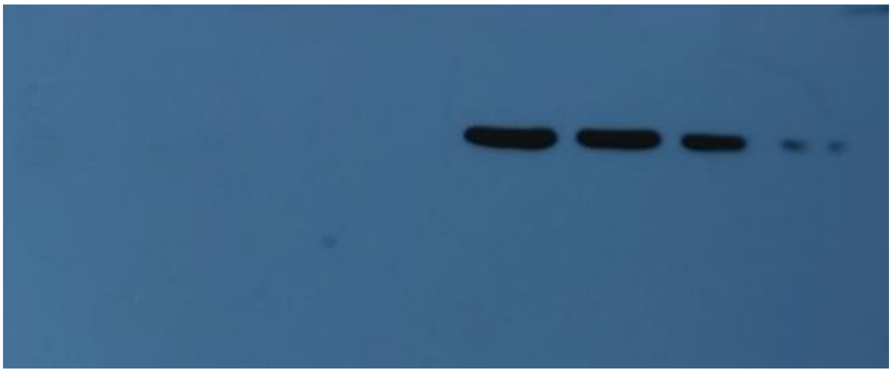

Bax

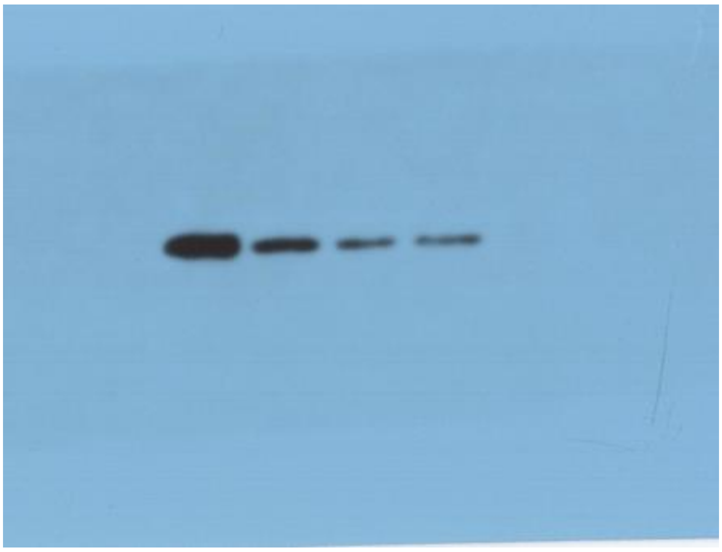

Caspase-3

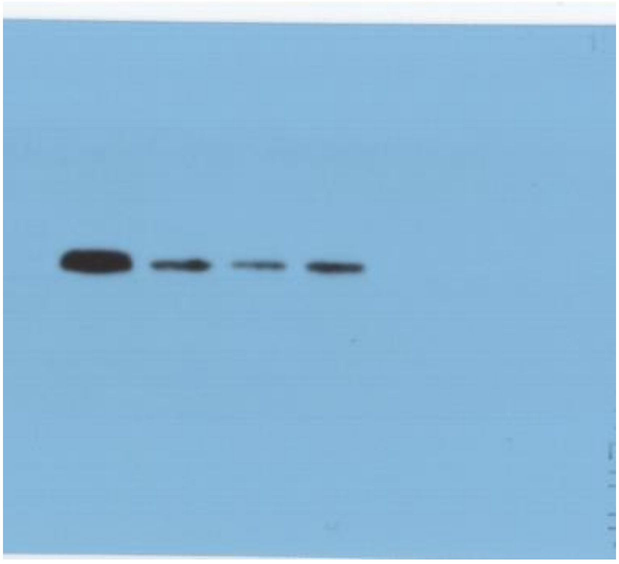

GAPDH

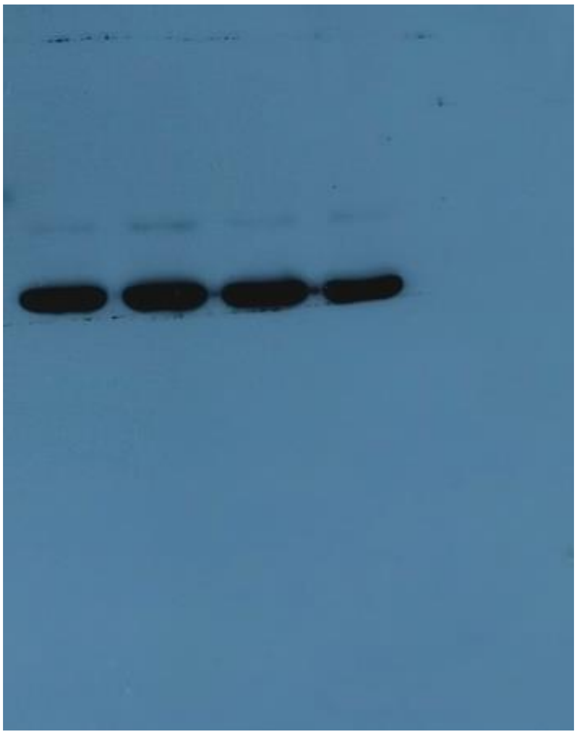

Figure. 2A

CTR1

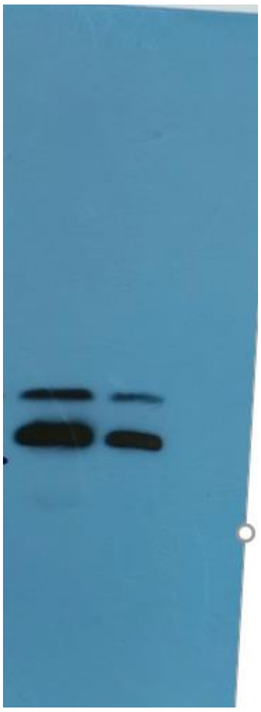

GAPDH

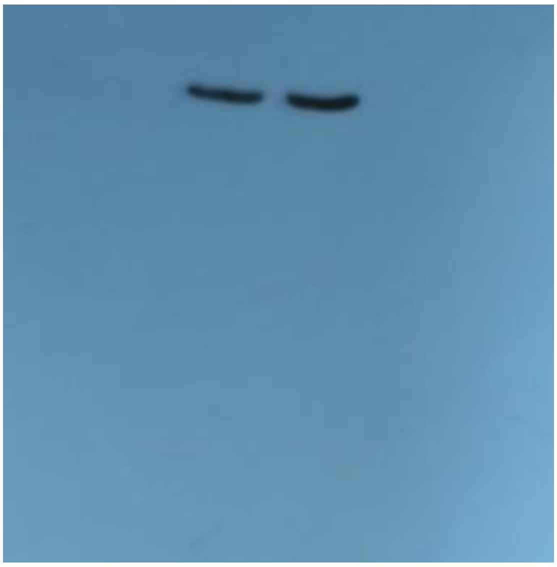

CTR1

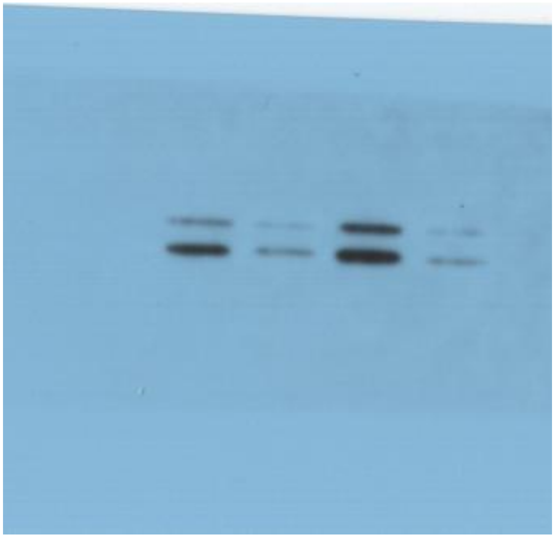

GAPDH

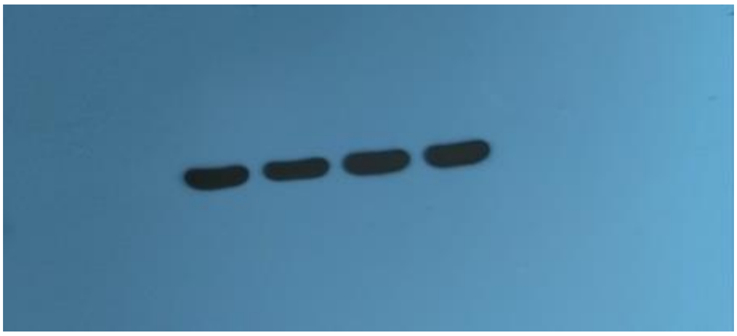

Figure. 2D（左图）

CTR1

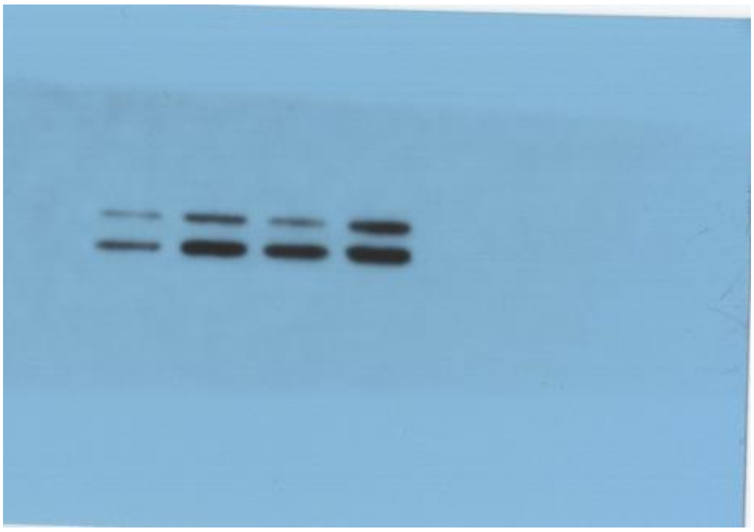

Bcl2

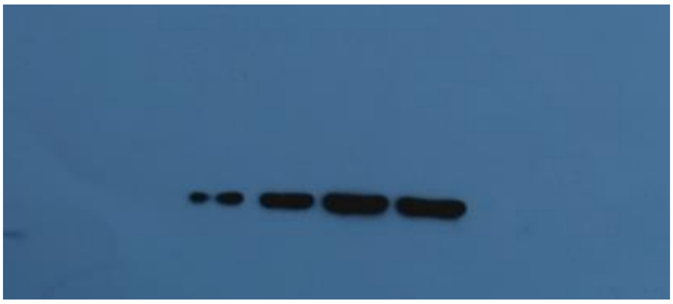

Bax

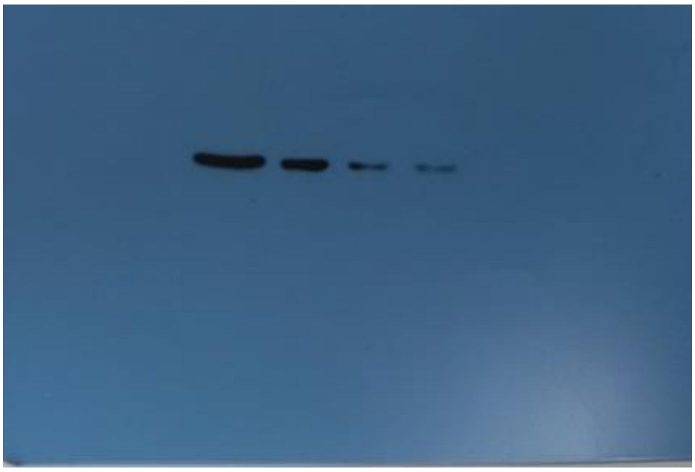

Caspase3

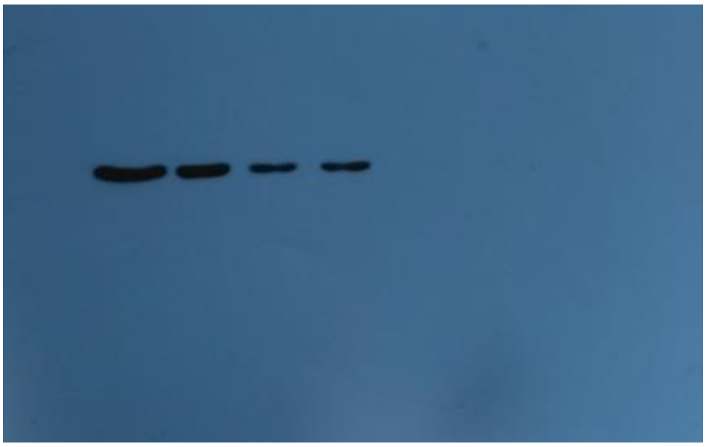

GAPDH

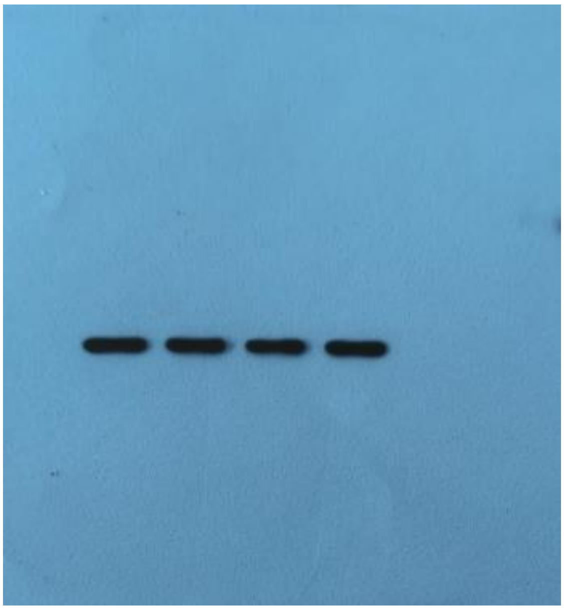

Figure. 2D（右图）
CTR1

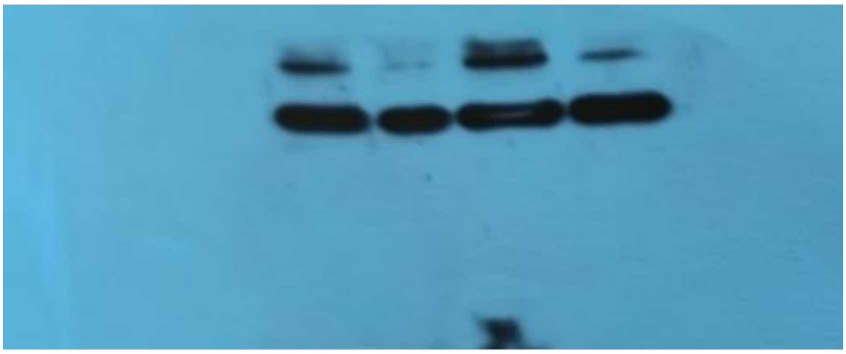

Bax

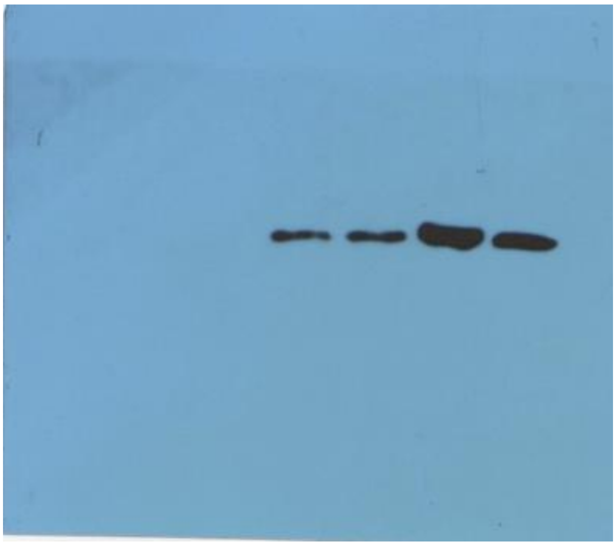

Caspase3

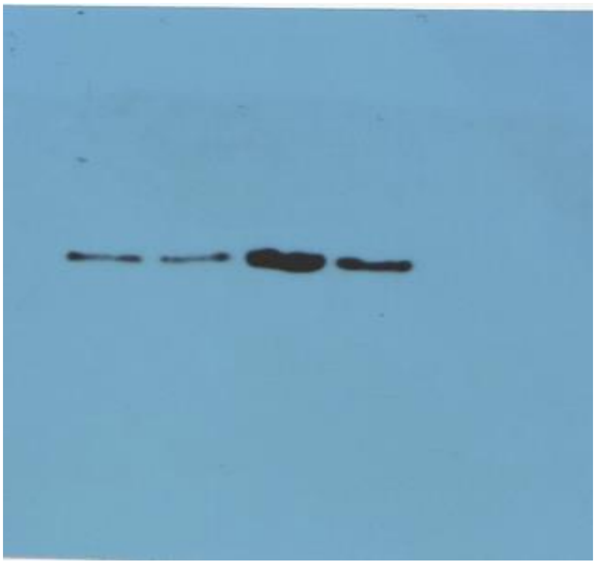

GAPDH

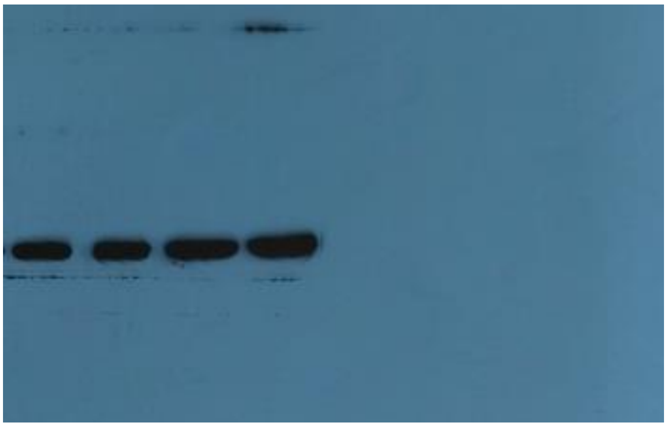

Figure. 3B（左图）

P53

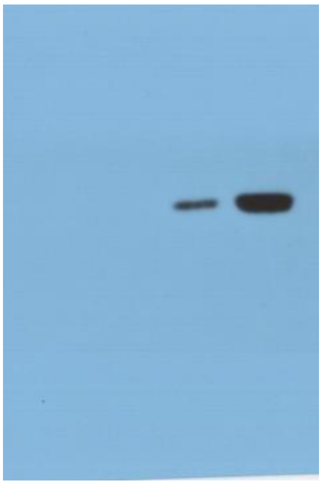

CTR1

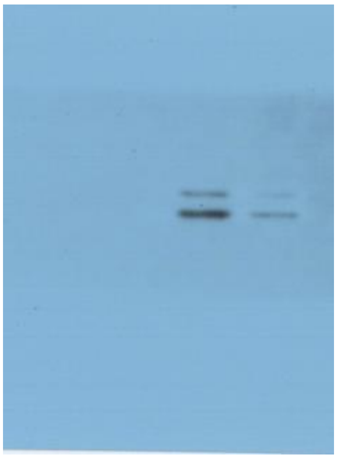

GAPDH

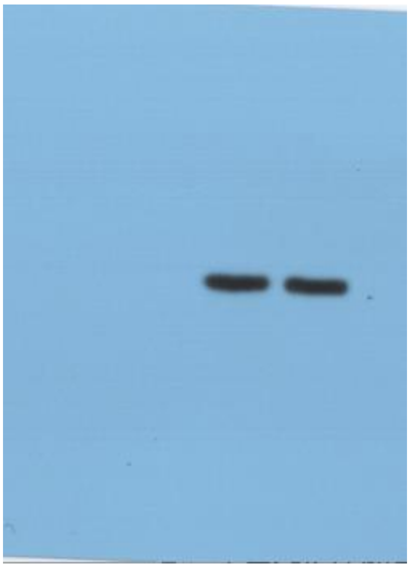

Figure. 3B（右图）
P53

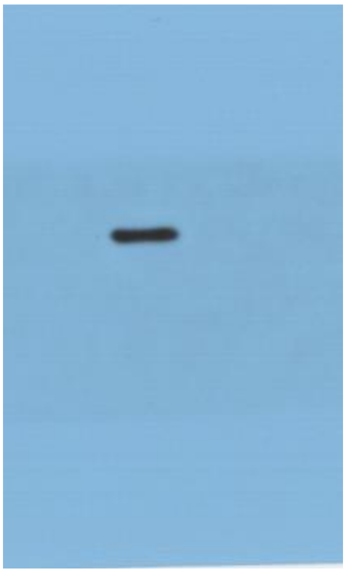

CTR1

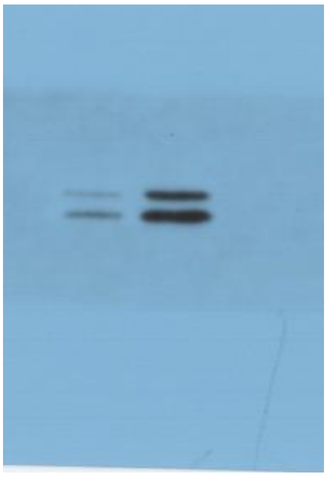

GAPDH

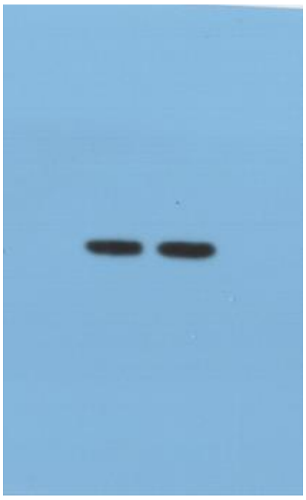

Figure. 3D（左图）

P53

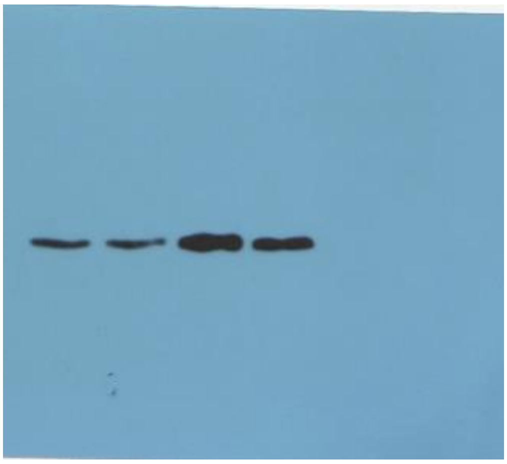

CTR1

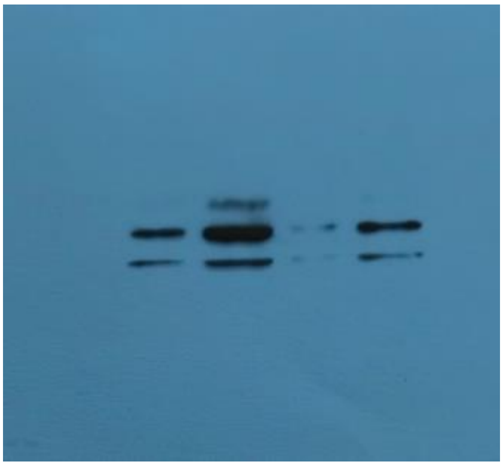

GAPDH

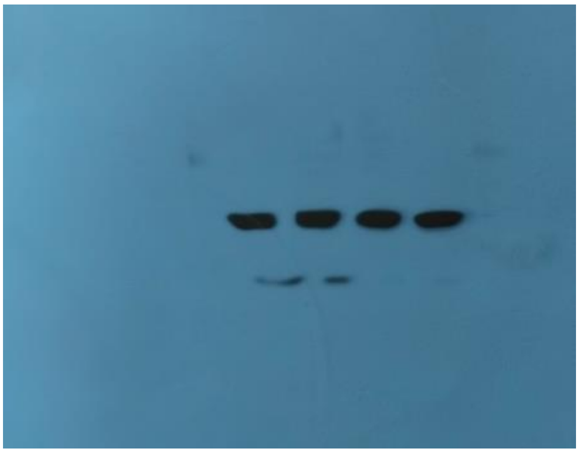

Figure. 3D（右图）

P53

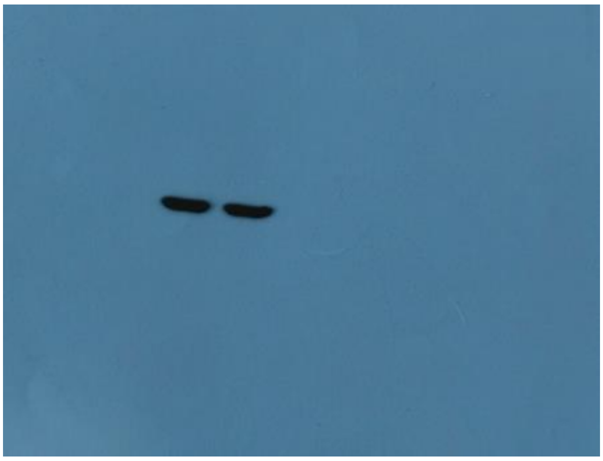

CTR1

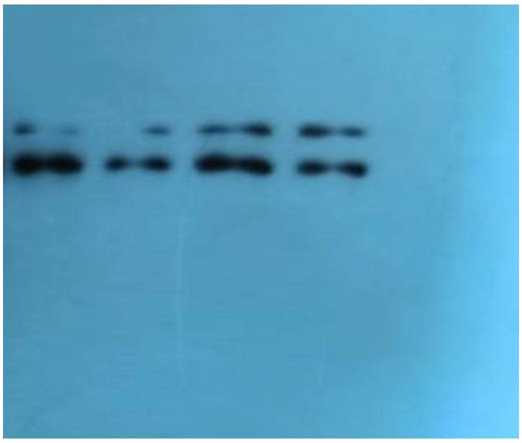

GAPDH

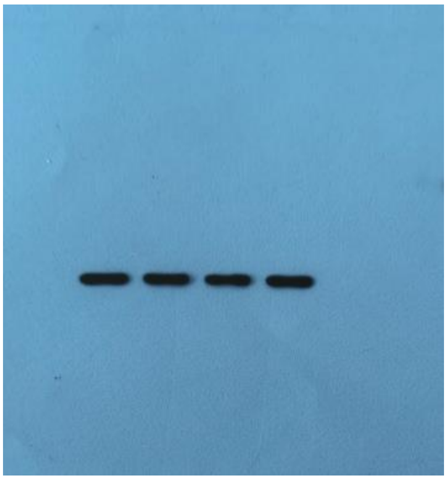

Figure. 4A

P53

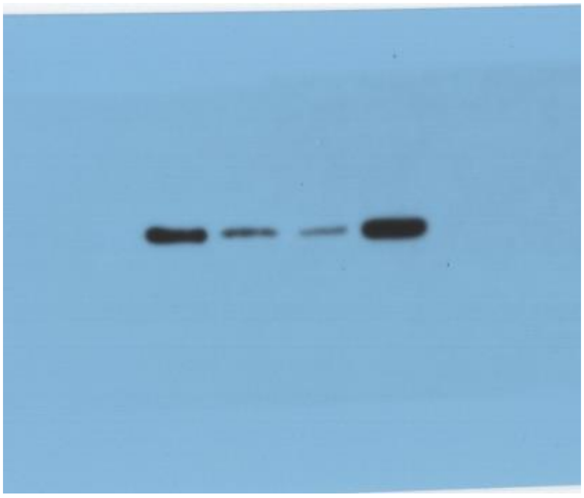

LaminA

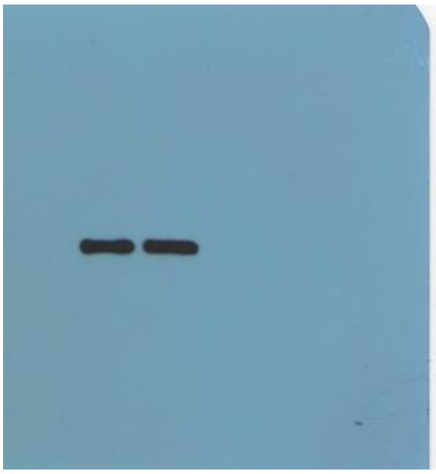

Tubulin

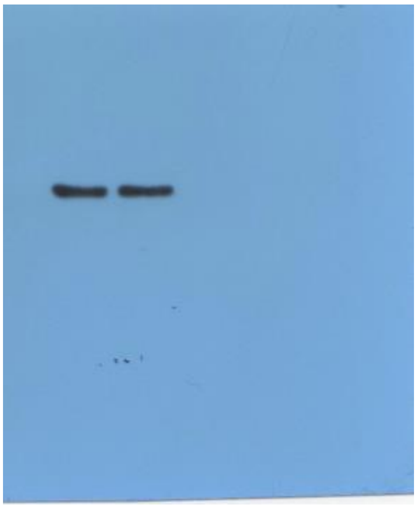

Figure. 5A（上图）
SP1

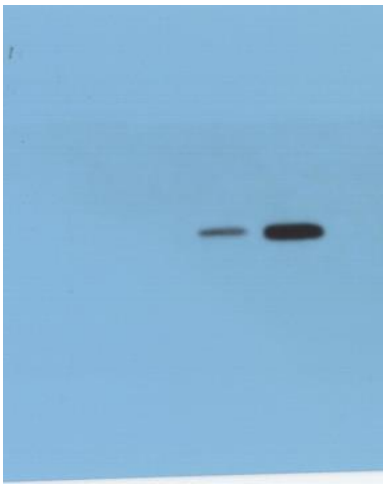

GPADH

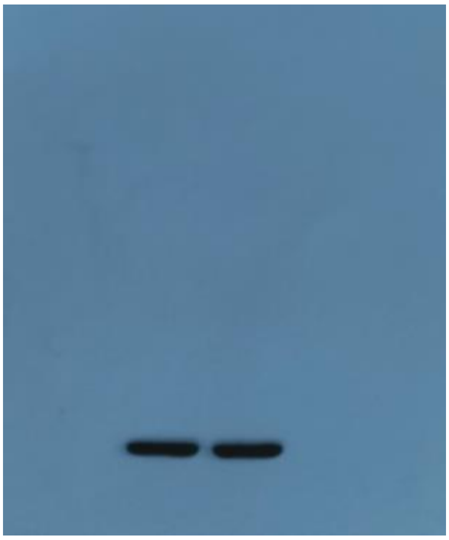

Figure. 5A（下图）
SP1

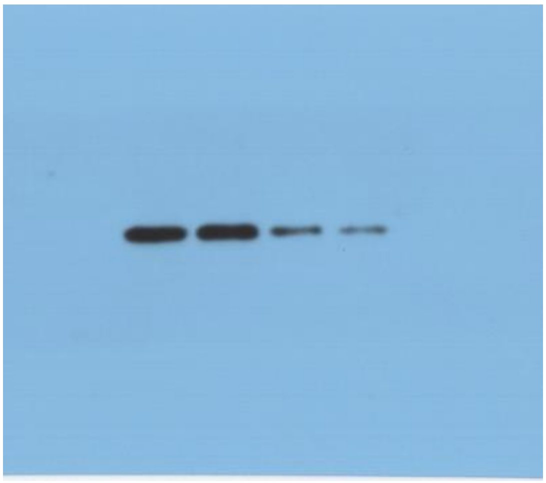

GAPDH

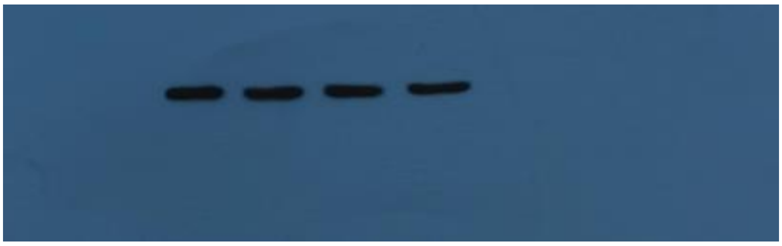

Figure. 5B（左图）

SP1

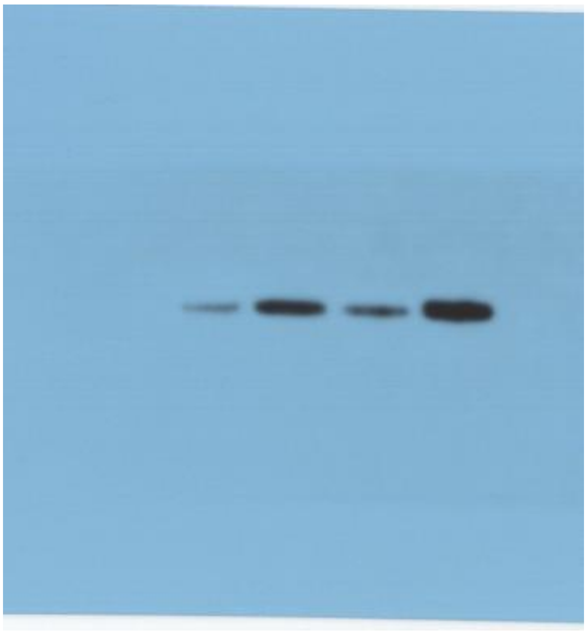

CTR1

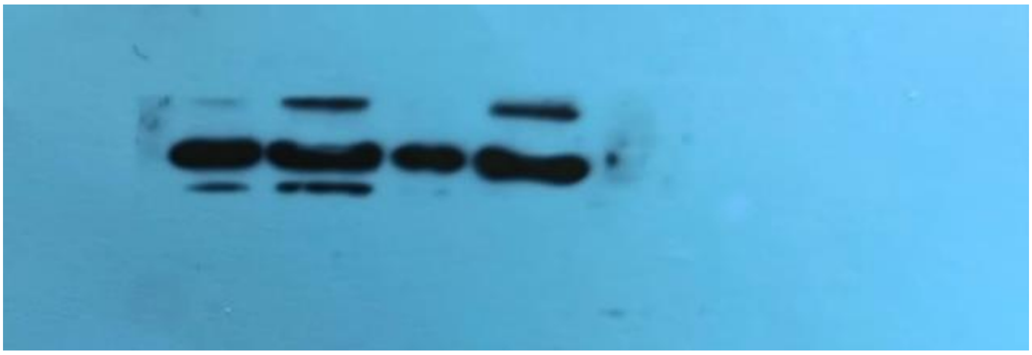

GAPDH

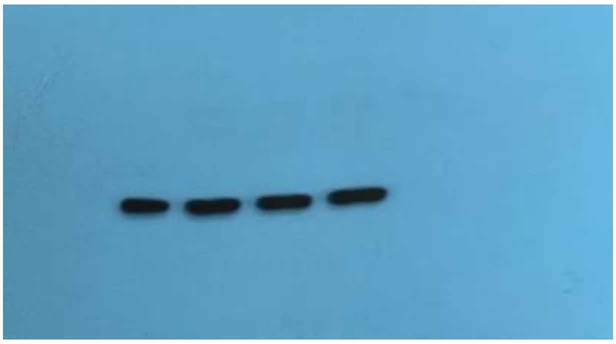

Figure. 5B（右图）

SP1

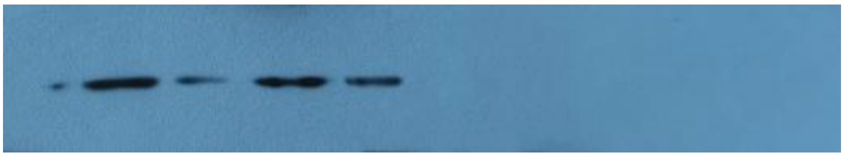

CTR1

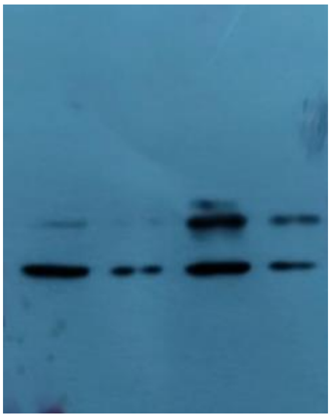

GAPDH

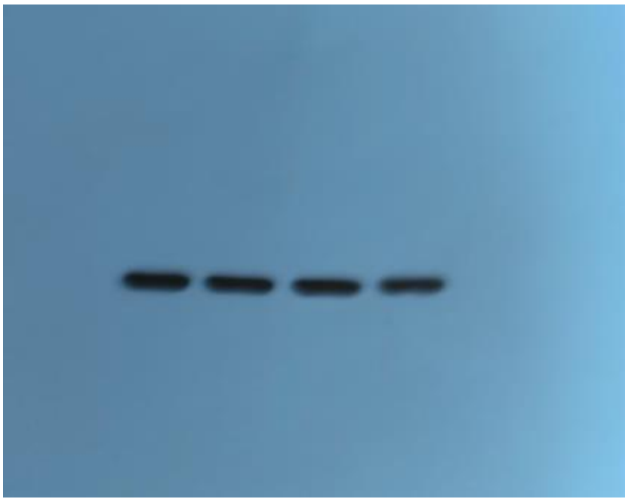

Figure. 5F

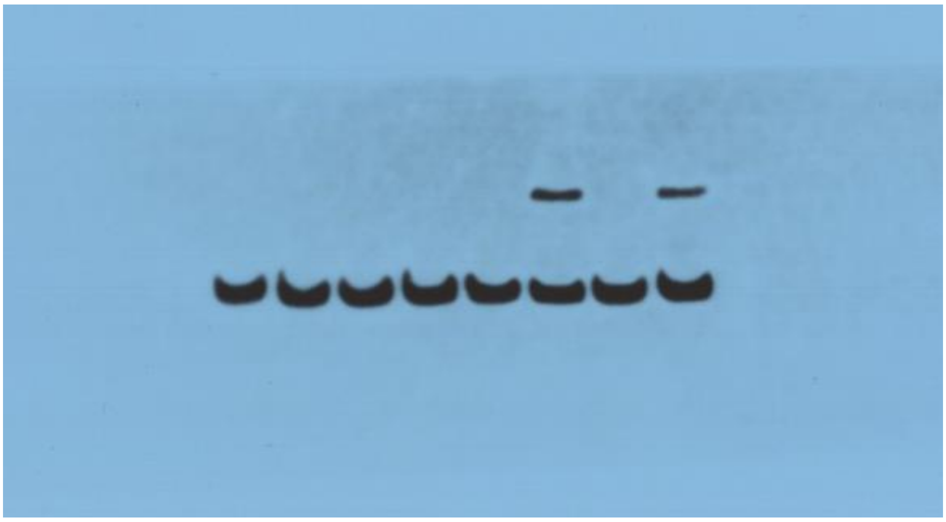

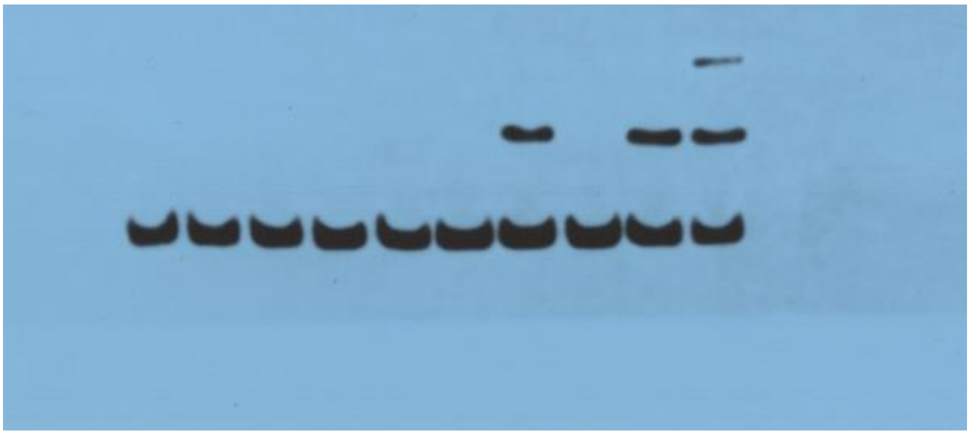

Figure. 6A（左图）
P53

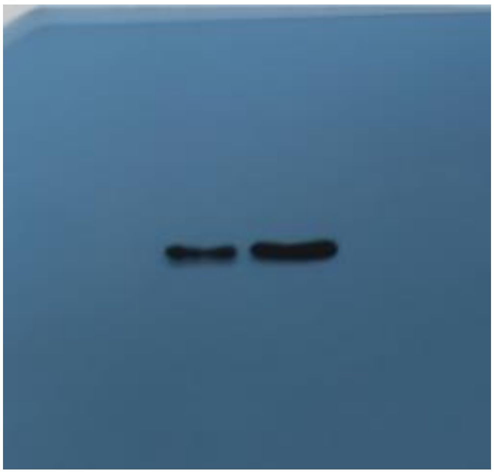

SP1

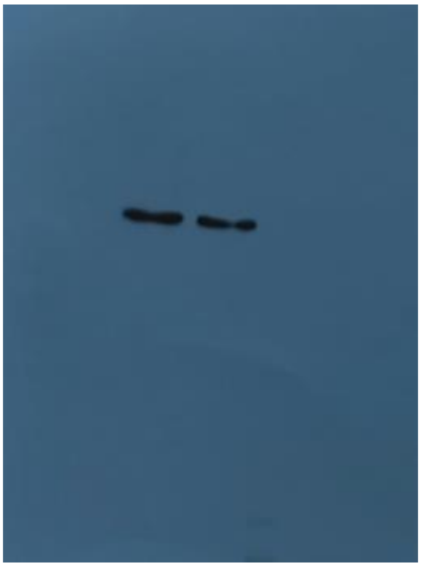

CTR1

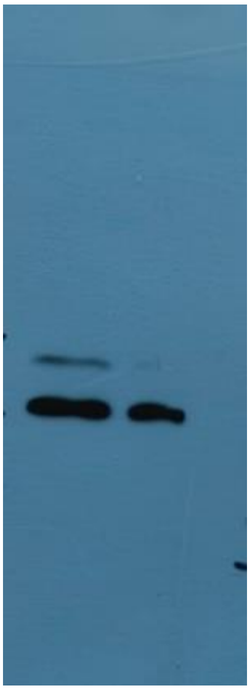

GAPDH

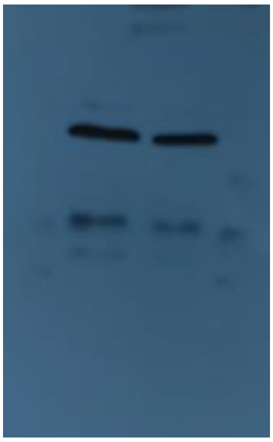

Figure. 6A（右图）

P53

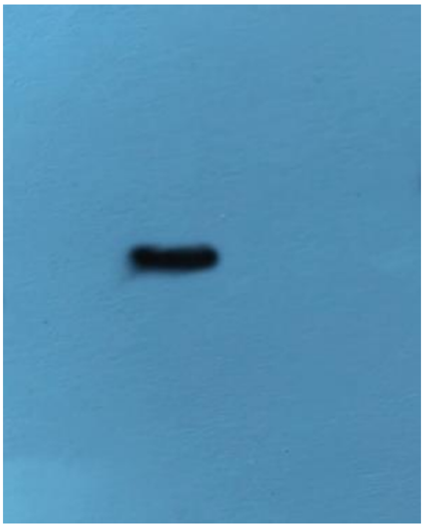

SP1

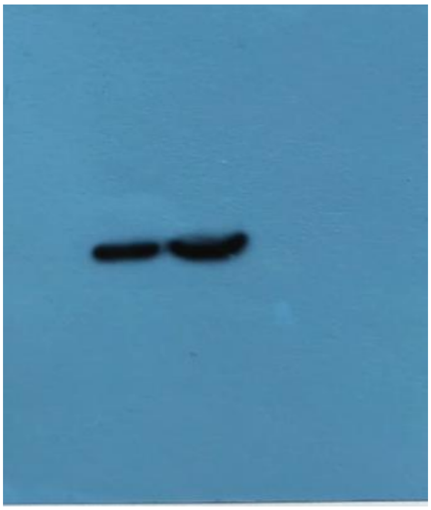

CTR1

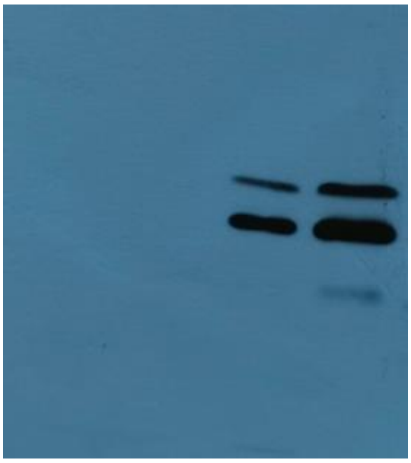

GAPDH

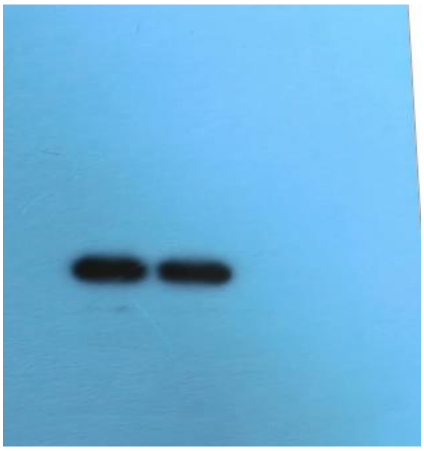

Figure. 6B（左图）

P53

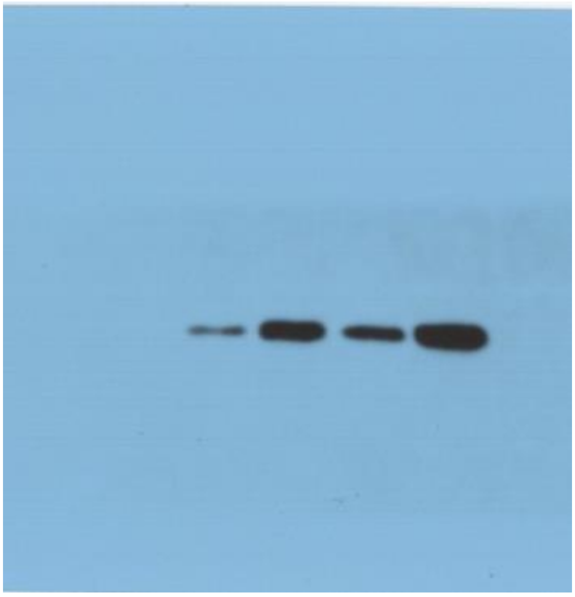

SP1

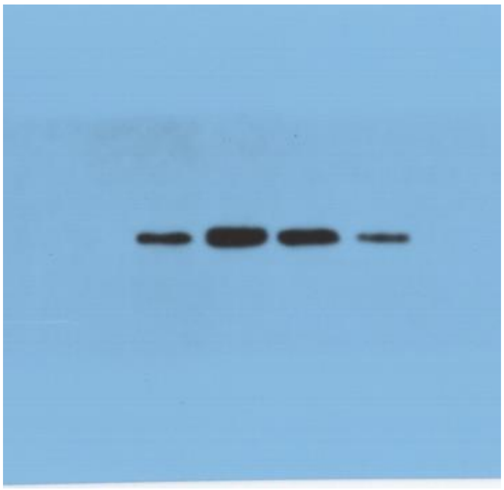

LaminA

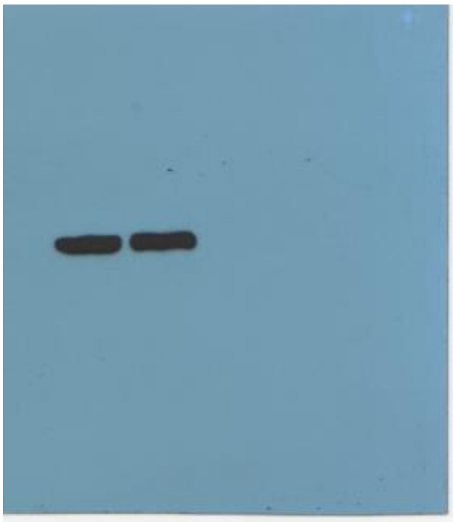

Tubulin

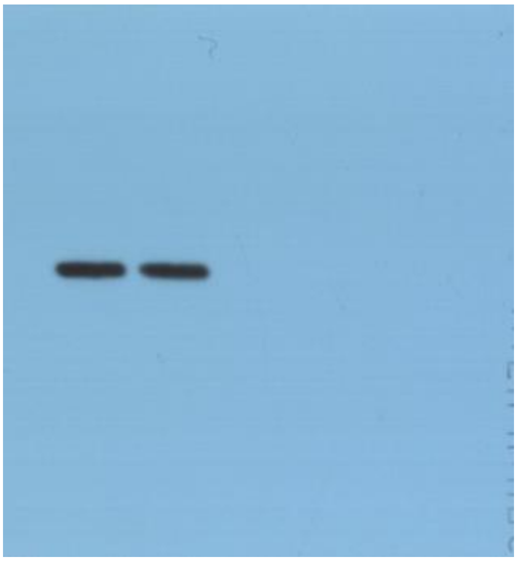

Figure. 6B（右图）
P53

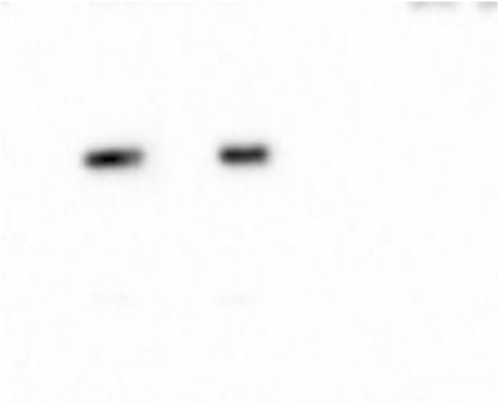

SP1

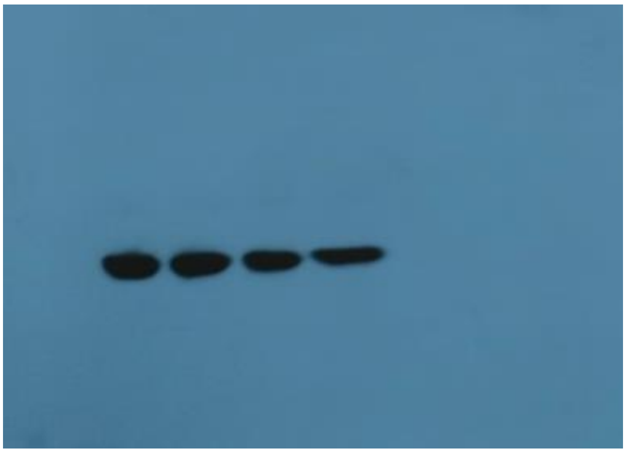

LaminA

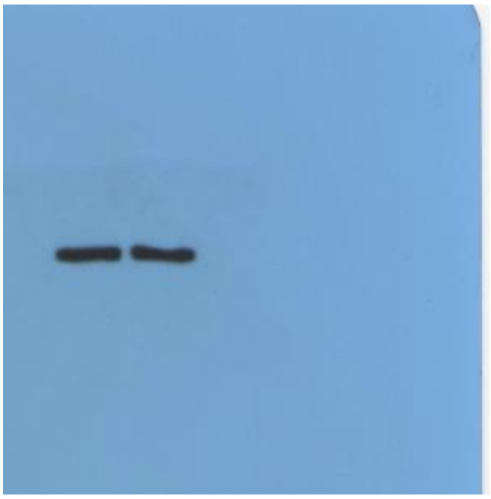

Tubulin

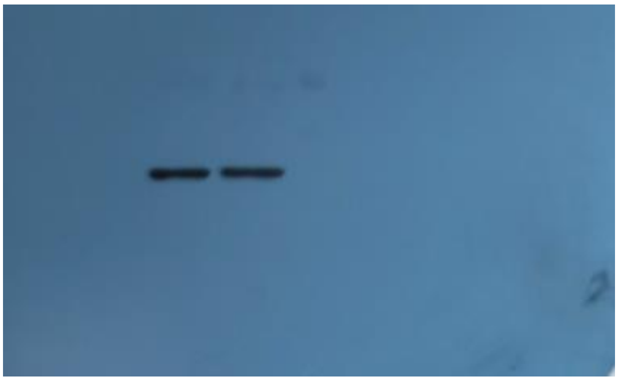

Figure. 6C
P53

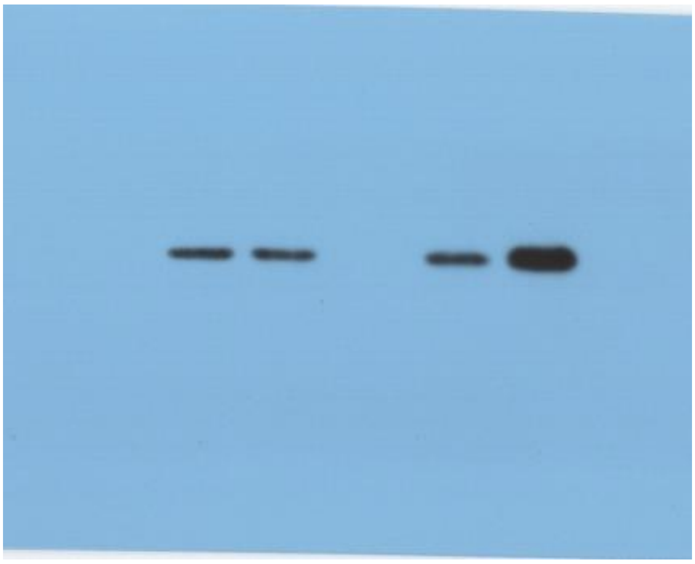

SP1

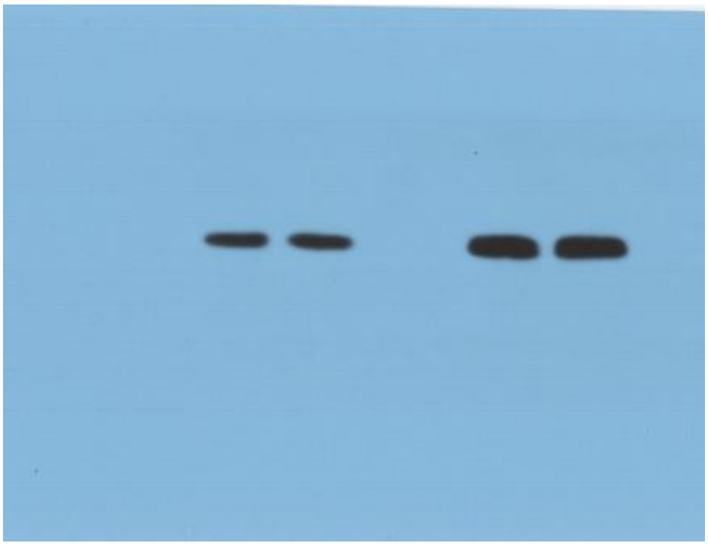

GAPDH

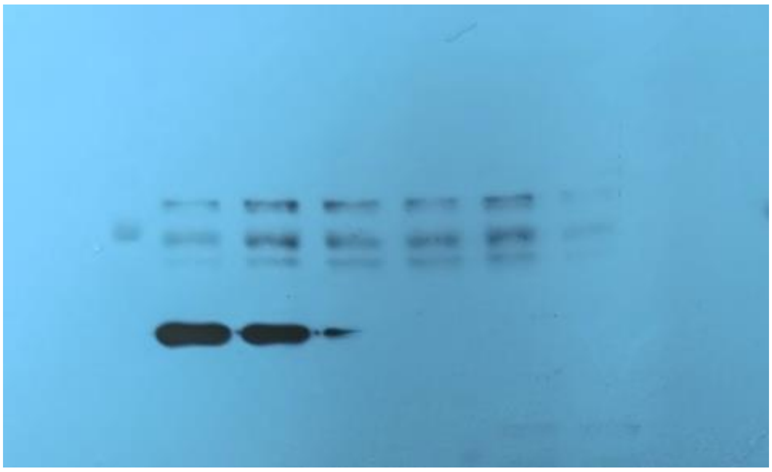

Supplement: Supplementary file 1 [file DataSheet_1.pdf]
