# Supplementary material for: p53 inhibits CTR1-mediated cisplatin absorption by suppressing SP1 nuclear translocation in osteosarcoma
Source: Front Oncol. 2023 Jan 26;12:1047194. doi: 10.3389/fonc.2022.1047194 (PMC9910081; doi:10.3389/fonc.2022.1047194)
Supplement: Supplementary file 2 [file Presentation_1.pptx]

## Slide 1
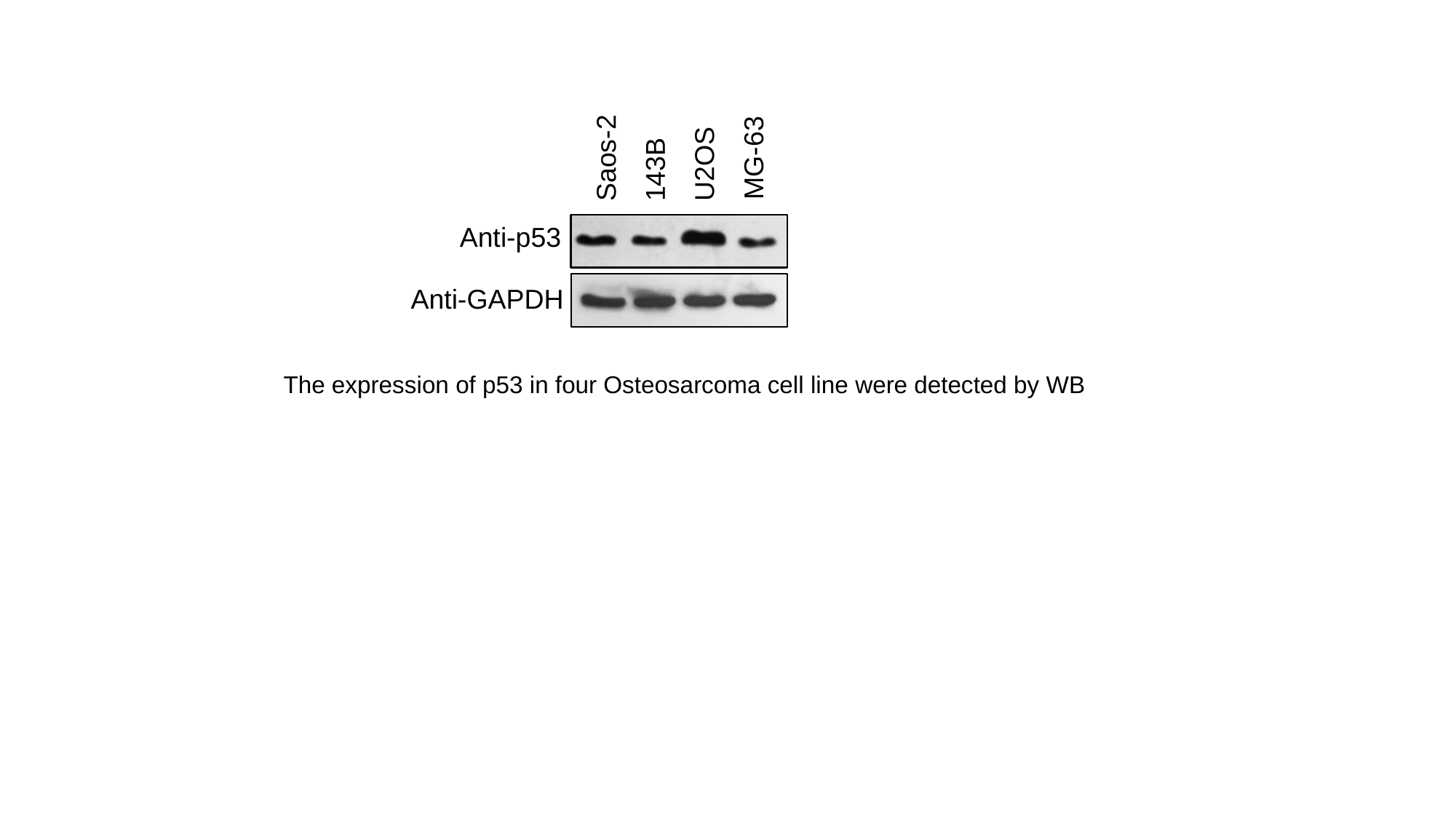

Saos-2
MG-63
U2OS
143B
Anti-p53
Anti-GAPDH
The expression of p53 in four Osteosarcoma cell line were detected by WB

## Slide 2
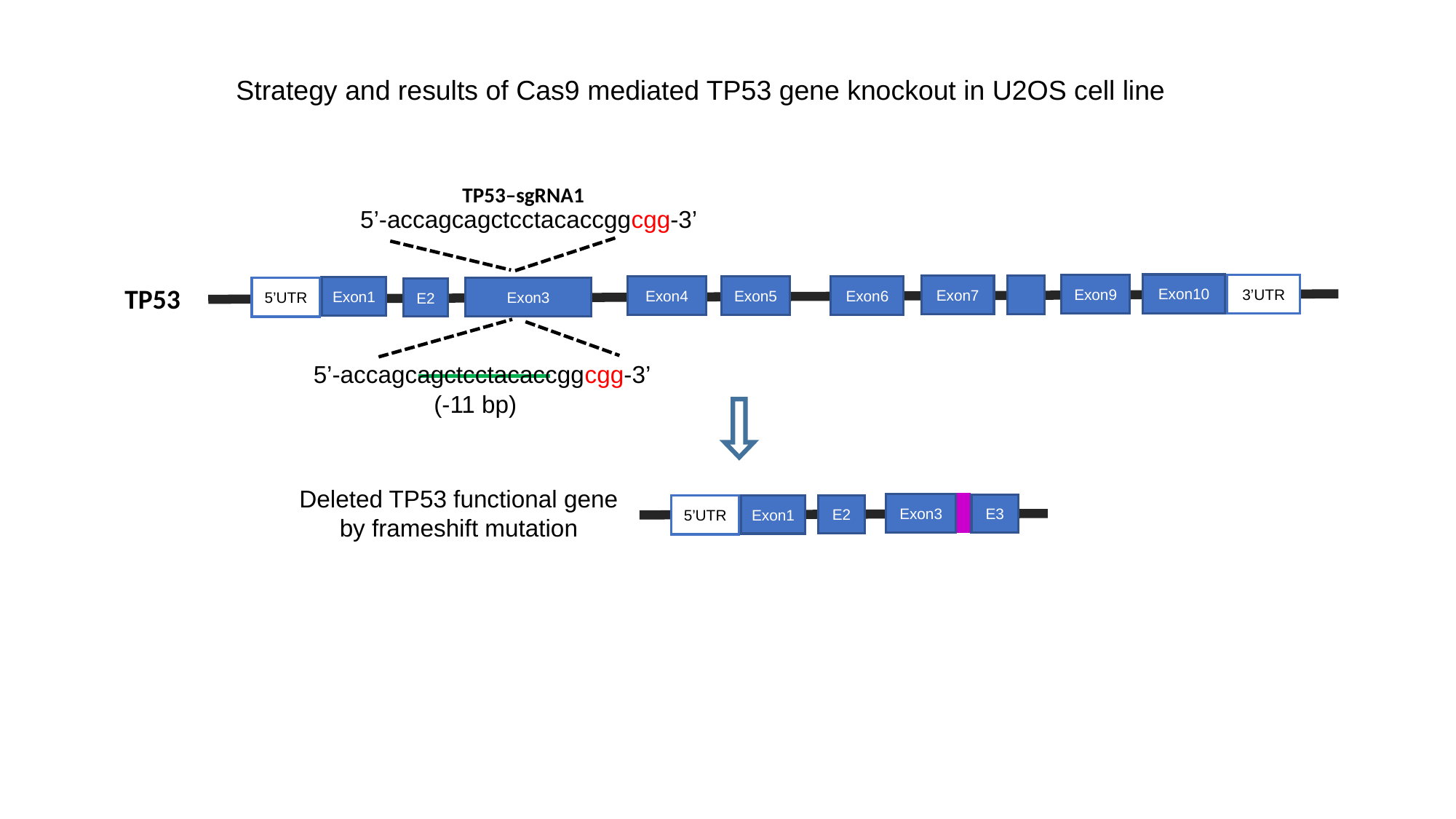

Strategy and results of Cas9 mediated TP53 gene knockout in U2OS cell line
TP53–sgRNA1
5’-accagcagctcctacaccggcgg-3’
Exon10
Exon9
3’UTR
Exon7
Exon6
Exon5
TP53
Exon4
Exon1
Exon3
5’UTR
E2
5’-accagcagctcctacaccggcgg-3’
(-11 bp)
Deleted TP53 functional gene by frameshift mutation
Exon3
E3
Exon1
E2
5’UTR

## Slide 3
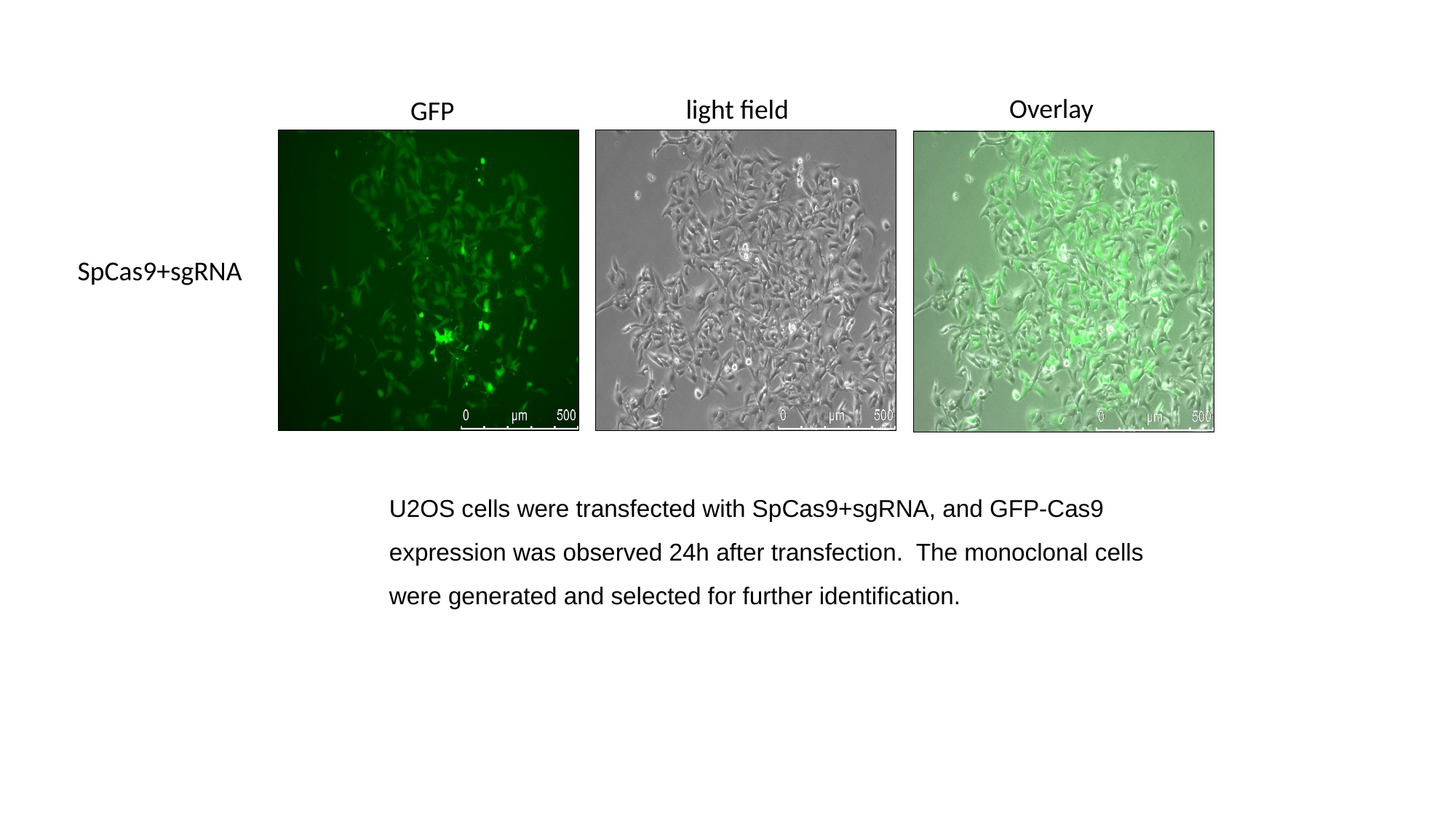

Overlay
light field
GFP
SpCas9+sgRNA
U2OS cells were transfected with SpCas9+sgRNA, and GFP-Cas9 expression was observed 24h after transfection. The monoclonal cells were generated and selected for further identification.

## Slide 4
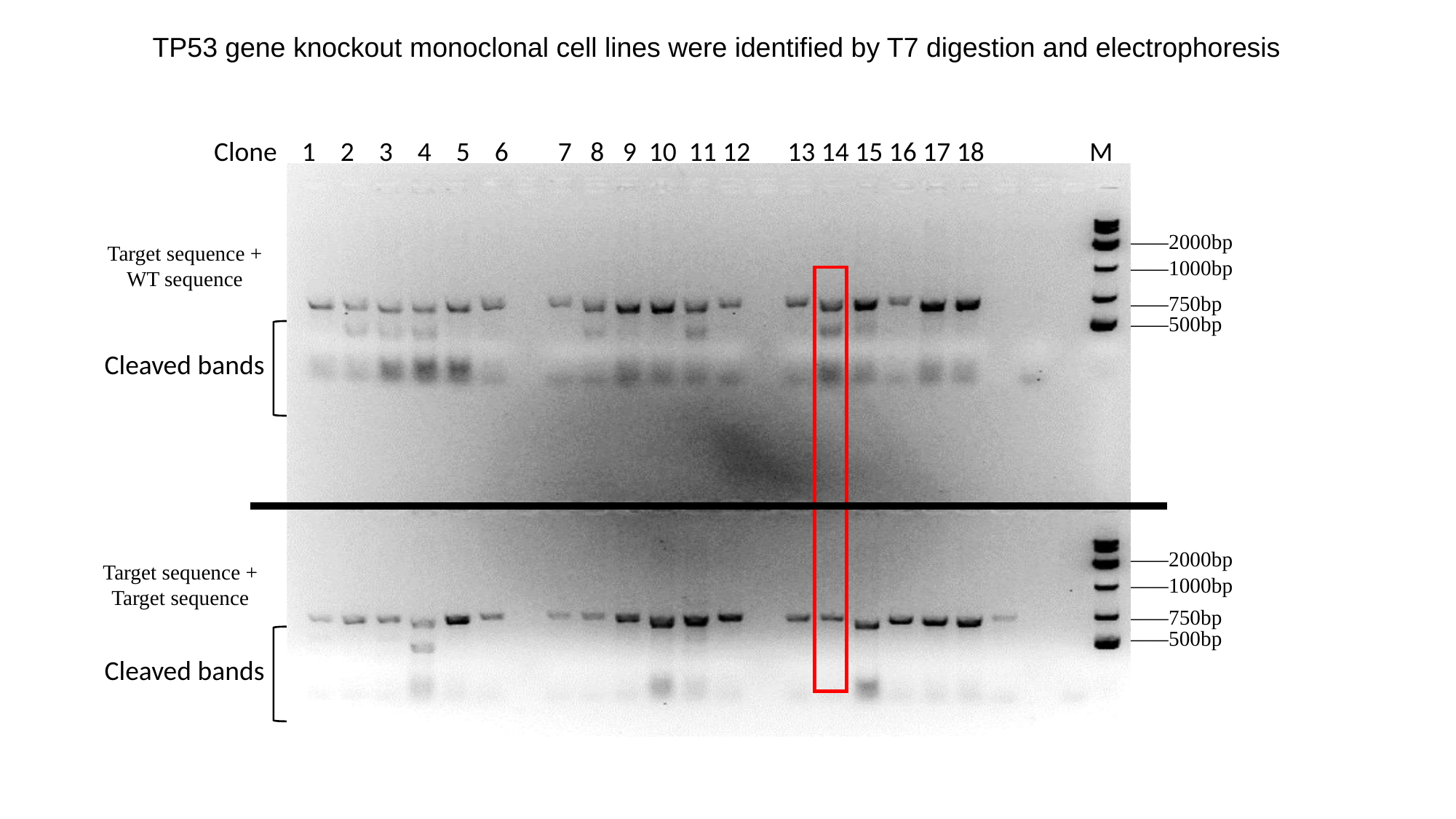

TP53 gene knockout monoclonal cell lines were identified by T7 digestion and electrophoresis
Clone 1 2 3 4 5 6 7 8 9 10 11 12 13 14 15 16 17 18 M
——2000bp
Target sequence +
WT sequence
——1000bp
——750bp
——500bp
Cleaved bands
——2000bp
Target sequence +
Target sequence
——1000bp
——750bp
——500bp
Cleaved bands

## Slide 5
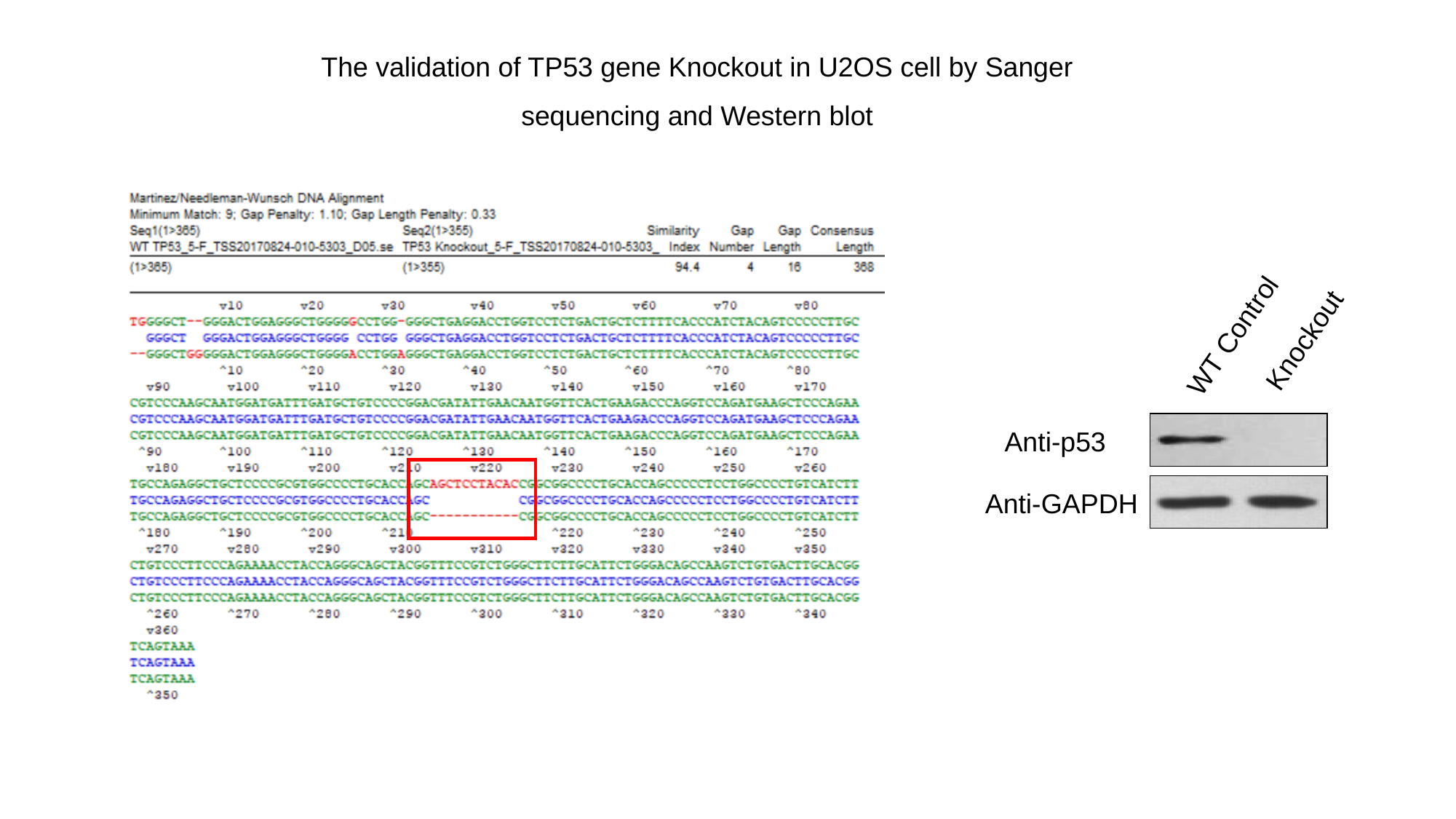

The validation of TP53 gene Knockout in U2OS cell by Sanger sequencing and Western blot
WT Control
Knockout
Anti-p53
Anti-GAPDH
